# Easy and efficient ensemble gene set testing with EGSEA

**DOI:** 10.12688/f1000research.12544.1

**Published:** 2017-11-14

**Authors:** Monther Alhamdoosh, Charity W. Law, Luyi Tian, Julie M. Sheridan, Milica Ng, Matthew E. Ritchie

**Affiliations:** 1 CSL Limited, Bio21 Institute, Parkville, Victoria, Australia; 2 Department of Medical Biology, The University of Melbourne, Parkville, Victoria, Australia; 3 Molecular Medicine Division, The Walter and Eliza Hall Institute of Medical Research, Parkville, Victoria, Australia; 4 Molecular Genetics of Cancer Division, The Walter and Eliza Hall Institute of Medical Research, Parkville, Victoria, Australia; 5 School of Mathematics and Statistics, The University of Melbourne, Parkville, Victoria, Australia

**Keywords:** gene expression, RNA-sequencing, microarrays, gene set enrichment, Bioconductor

## Abstract

Gene set enrichment analysis is a popular approach for prioritising the biological processes perturbed in genomic datasets. The Bioconductor project hosts over 80 software packages capable of gene set analysis. Most of these packages search for enriched signatures amongst differentially regulated genes to reveal higher level biological themes that may be missed when focusing only on evidence from individual genes. With so many different methods on offer, choosing the best algorithm and visualization approach can be challenging. The EGSEA package solves this problem by combining results from up to 12 prominent gene set testing algorithms to obtain a consensus ranking of biologically relevant results.This workflow demonstrates how EGSEA can extend limma-based differential expression analyses for RNA-seq and microarray data using experiments that profile 3 distinct cell populations important for studying the origins of breast cancer. Following data normalization and set-up of an appropriate linear model for differential expression analysis, EGSEA builds gene signature specific indexes that link a wide range of mouse or human gene set collections obtained from MSigDB, GeneSetDB and KEGG to the gene expression data being investigated. EGSEA is then configured and the ensemble enrichment analysis run, returning an object that can be queried using several S4 methods for ranking gene sets and visualizing results via heatmaps, KEGG pathway views, GO graphs, scatter plots and bar plots. Finally, an HTML report that combines these displays can fast-track the sharing of results with collaborators, and thus expedite downstream biological validation. EGSEA is simple to use and can be easily integrated with existing gene expression analysis pipelines for both human and mouse data.

## Introduction

Gene set enrichment analysis allows researchers to efficiently extract biological insights from long lists of differentially expressed genes by interrogating them at a systems level. In recent years, there has been a proliferation of gene set enrichment (GSE) analysis methods released through the Bioconductor project ^1^ together with a steady increase in the number of gene set collections available through online databases such as MSigDB ^2^, GeneSetDB ^3^ and KEGG ^4^. In an effort to unify these computational methods and knowledge-bases, the **EGSEA** R/Bioconductor package was developed. EGSEA, which stands for *Ensemble of Gene Set Enrichment Analyses* ^5^ combines the results from multiple algorithms to arrive at a consensus gene set ranking to identify biological themes and pathways perturbed in an experiment. EGSEA calculates seven statistics to combine the individual gene set statistics of base GSE methods to rank biologically relevant gene sets. The current version of the **EGSEA** package ^6^ utilizes the analysis results of up to twelve prominent GSE algorithms that include: ***ora*** ^7^, ***globaltest*** ^8^, ***plage*** ^9^, ***safe*** ^10^, ***zscore*** ^11^, ***gage*** ^12^, ***ssgsea*** ^13^, ***padog*** ^14^, ***gsva*** ^15^, ***camera*** ^16^, ***roast*** ^17^ and ***fry*** ^17^. The ***ora***, ***gage***, ***camera*** and ***gsva*** methods depend on a *competitive* null hypothesis which assumes the genes in a set do not have a stronger association with the experimental condition compared to randomly chosen genes outside the set. The remaining eight methods are based on a *self-contained* null hypothesis that only considers genes within a set and again assumes that they have no association with the experimental condition.

EGSEA provides access to a diverse range of gene signature collections through the **EGSEAdata** package that includes more than 25,000 gene sets for human and mouse organised according to their database sources ( Table 1). For example, MSigDB ^2^ includes a number of collections (Hallmark (h) and c1–c7) that explore different biological themes ranging from very broad (h, c2, c5) through to more specialised ones focusing on cancer (c4, c6) and immunology (c7). The other main sources are GeneSetDB ^3^ and KEGG ^4^ which have similar collections focusing on different biological characteristics ( Table 1). The choice of collection/s in any given analysis should of course be guided by the biological question of interest. The MSigDB c2 and c5 collections are the most widely used in our own analysis practice, spanning a wide range of biological processes and can often reveal new biological insights when applied to a given dataset.

**Table 1.** Summary of the gene set collections available in the EGSEAdata package.

Database	Collection	Description
**MSigDB**	h Hallmarks c1 Positional c2 Curated c3 Motif c4 Computational c5 GO c6 Oncogenic c7 Immunologic	Gene sets representing well-defined biological states or processes that have coherent expression. Gene sets by chromosome and cytogenetic band. Gene sets obtained from a variety of sources, including online pathway databases and the biomedical literature. Gene sets of potential targets regulated by transcription factors or microRNAs. Gene sets defined computationally by mining large collections of cancer-oriented microarray data. Gene sets annotated by Gene Ontology (GO) terms. Gene sets of the major cellular pathways disrupted in cancer. Gene sets representing different cell types and stimulations relevant to the immune system.
**KEGG**	Signalling Disease Metabolic	Gene sets obtained from the KEGG database.
**GeneSetDB**	Pathway Disease Drug Regulation GO Terms	Gene sets obtained from various online databases.

**Figure 1.**
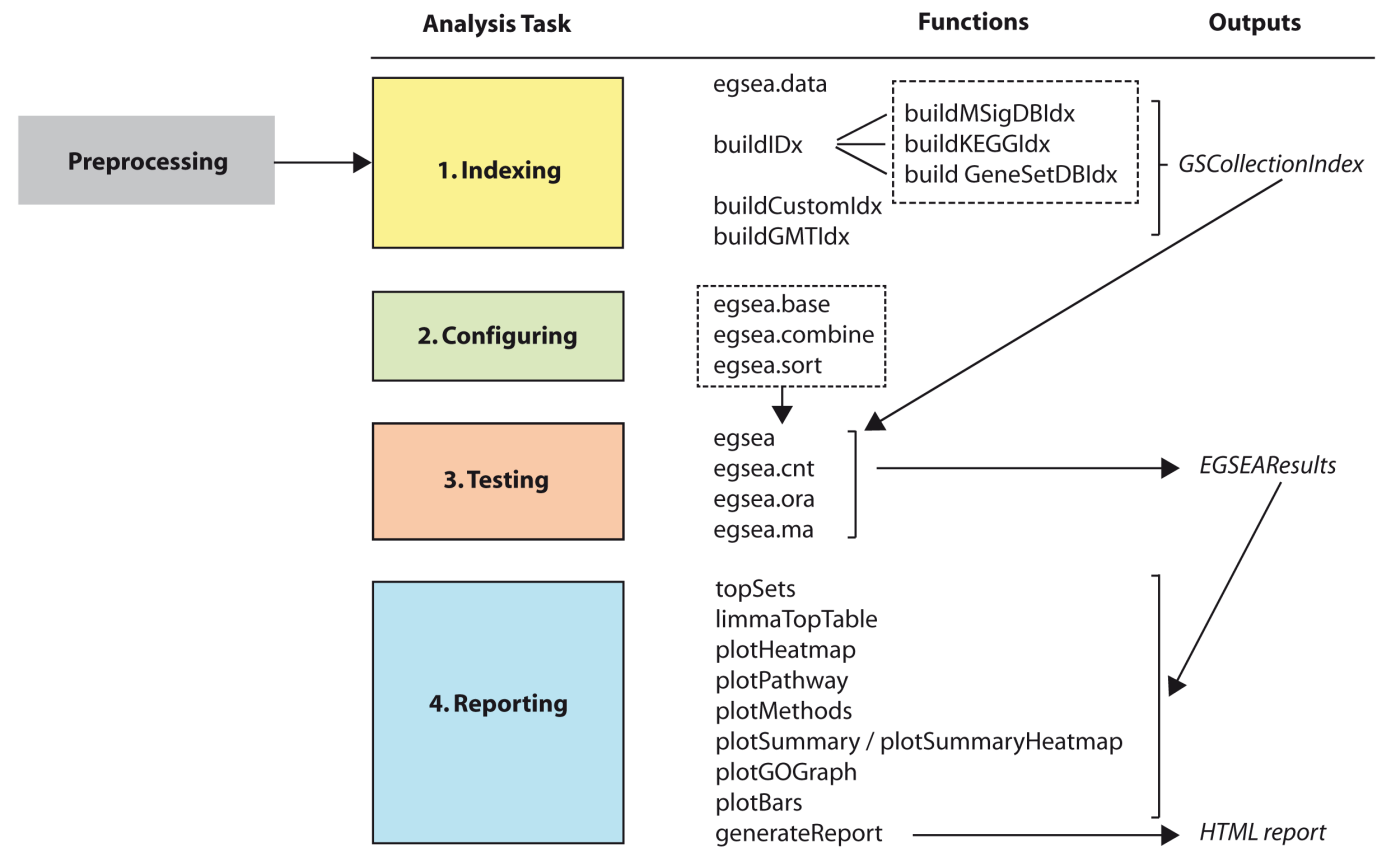
The main steps in an EGSEA analysis and the functions that perform each task.

The purpose of this article is to demonstrate the gene set testing workflow available in **EGSEA** on both RNA-seq and microarray data. Each analysis involves four major steps that are summarized in Figure 1: (1) selecting appropriate gene set collections for analysis and building an index that maps between the members of each set and the expression matrix; (2) choosing the base GSE methods to combine and the ranking options; (3) running the EGSEA test and (4) reporting results in various ways to share with collaborators. The **EGSEA** functions involved in each of these steps are introduced with code examples to demonstrate how they can be deployed as part of a limma differential expression analysis to help with the interpretation of results.

## Gene expression profiling of the mouse mammary gland

The first experiment analysed in this workflow is an RNA-seq dataset from Sheridan *et al.* (2015) ^18^ that consists of 3 cell populations (Basal, Luminal Progenitor (LP) and Mature Luminal (ML)) sorted from the mammary glands of female virgin mice. Triplicate RNA samples from each population were obtained in 3 batches and sequenced on an Illumina HiSeq 2000 using a 100 base-pair single-ended protocol. Raw sequence reads from the fastq files were aligned to the mouse reference genome (mm10) using the **Rsubread** package ^19^. Next, gene-level counts were obtained using featureCounts ^20^ based on **Rsubread’s** built-in *mm10* RefSeq-based annotation. The raw data along with further information on experimental design and sample preparation can be downloaded from the Gene Expression Omnibus (GEO, www.ncbi.nlm.nih.gov/geo/) using GEO Series accession number GSE63310 and will be preprocessed according to the RNA-seq workflow published by Law *et al.* (2016) ^21^.

The second experiment analysed in this workflow comes from Lim *et al.* (2010) ^22^ and is the microarray equivalent of the RNA-seq dataset mentioned above. The same 3 populations (Basal (also referred to as “MaSC-enriched”), LP and ML) were sorted from mouse mammary glands via flow cytometry. Total RNA from 5 replicates of each cell population were hybridised onto 3 Illumina MouseWG-6 v2 BeadChips. The intensity files and chip annotation file available in Illumina’s proprietary formats (IDAT and BGX respectively) can be downloaded from http://bioinf.wehi.edu.au/EGSEA/arraydata.zip. The raw data from this experiment is also available from GEO under Series accession number GSE19446.

## Analysis of RNA-seq data with EGSEA

Our RNA-seq analysis follows on directly from the workflow of Law *et al.* (2016) which performs a differential gene expression analysis on this data set using the Bioconductor packages **edgeR** ^23^, **limma** ^24^ and **Glimma** ^25^ with gene annotation from the **Mus.musculus** package ^26^. The **limma** package offers a well-developed suite of statistical methods for dealing with differential expression for both microarray and RNA-seq datasets and will be used in the analyses of both datasets presented in this workflow.

### Reading, preprocessing and normalisation of RNA-seq data

To get started with this analysis, download the R data file from http://bioinf.wehi.edu.au/EGSEA/mam.rnaseq.rdata. The code below loads the preprocessed count matrix from Law *et al.* (2016), performs TMM normalisation ^27^ on the raw counts, and calculates voom weights for use in comparisons of gene expression between Basal and LP, Basal and ML, and LP and ML populations.



                        > library(limma)
> library(edgeR)
> load("mam.rnaseq.rdata")
> names(mam.rnaseq.data)
[1] "samples" "counts"  "genes"
> dim(mam.rnaseq.data)
[1] 14165     9
> x = calcNormFactors(mam.rnaseq.data, method = "TMM")
> design = model.matrix(~0+x$samples$group+x$samples$lane)
> colnames(design) = gsub("x\\$samples\\$group", "", colnames(design))
> colnames(design) = gsub("x\\$samples\\$lane", "", colnames(design))
> head(design) 
  Basal LP ML L006 L008	 
1     0  1  0    0    0	 
2     0  0  1    0    0	 
3     1  0  0    0    0	 
4     1  0  0    1    0	 
5     0  0  1    1    0	 
6     0  1  0    1    0	 
> contr.matrix = makeContrasts(
+	  BasalvsLP = Basal-LP,
+	  BasalvsML = Basal - ML,
+	  LPvsML = LP - ML,
+	  levels = colnames(design))
> head(contr.matrix)
       Contrasts 
Levels  BasalvsLP BasalvsML LPvsML	 
  Basal	        1	  1	 0	 
  LP	       -1	  0	 1	 
  ML	        0	 -1	-1	 
  L006	        0	  0	 0	 
  L008	        0	  0	 0
                    


The voom function ^28^ from the **limma** package converts counts to log-counts-per-million (log-cpm) and calculates observation-level precision weights. The ***voom*** object ( v) contains normalized log-cpm values and gene information used by all of the methods in the EGSEA analysis below. The precision weights stored within v are also used by the ***camera***, ***roast*** and ***fry*** gene set testing methods.



                        > v = voom(x, design, plot=FALSE)

                        > names(v)

                        [1] "genes"   "targets" "E"       "weights" "design"
                    


For further information on preprocessing see Law *et al.* (2016), as a detailed explanation of these steps is beyond the scope of this article.

## Gene set testing

The EGSEA algorithm makes use of the ***voom*** object ( v), a design matrix ( design) and an optional contrasts matrix ( contr.matrix). The design matrix describes how the samples in the experiment relate to the coefficients estimated by the linear model ^29^. The contrasts matrix then compares two or more of these coefficients to allow relative assessment of differential expression. Base methods that utilize linear models such as those from **limma** and **GSVA** ( ***gsva***, ***plage***, ***zscore*** and ***ssgsea***) make use of the design and contrasts matrices directly. For methods that do not support linear models, these two matrices are used to extract the group information for each comparison.

### 1. Exploring, selecting and indexing gene set collections

The package **EGSEAdata** includes more than 25,000 gene sets organized in collections depending on their database sources. Summary information about the gene set collections available in **EGSEAdata** can be displayed as follows:



                        > library(EGSEAdata)
> egsea.data("mouse")
The following databases are available in EGSEAdata for Mus  musculus:


                        Database name: KEGG Pathways
Version: NA
Download/update date: 07 March 2017
Data source: gage::kegg.gsets()
Supported species: human, mouse, rat
Gene set collections: Signaling, Metabolism, Disease
Related data objects: kegg.pathways
Number of gene sets in each collection for Mus  musculus :
Signaling: 132
Metabolism: 89
Disease: 67


                        Database name: Molecular Signatures Database (MSigDB)
Version: 5.2
Download/update date: 07 March 2017
Data source: http://software.broadinstitute.org/gsea
Supported species: human, mouse
Gene set collections: h, c1, c2, c3, c4, c5, c6, c7
Related data objects: msigdb, Mm.H, Mm.c2, Mm.c3, Mm.c4, Mm.c5, Mm.c6, Mm.c7
Number of gene sets in each collection for  Mus musculus :
h Hallmark Signatures: 50
c2 Curated Gene Sets: 4729
c3 Motif Gene Sets: 836
c4 Computational Gene Sets: 858
c5 GO Gene Sets: 6166
c6 Oncogenic Signatures: 189
c7 Immunologic Signatures: 4872


                        Database name: GeneSetDB Database
Version: NA
Download/update date: 15 January 2016
Data source: http://www.genesetdb.auckland.ac.nz/
Supported species: human, mouse, rat
Gene set collections: gsdbdis, gsdbgo, gsdbdrug, gsdbpath, gsdbreg
Related data objects: gsetdb.human, gsetdb.mouse, gsetdb.rat
Number of gene sets in each collection for  Mus musculus :
GeneSetDB Drug/Chemical: 6019
GeneSetDB Disease/Phenotype: 5077
GeneSetDB Gene Ontology: 2202
GeneSetDB Pathway: 1444
GeneSetDB Gene Regulation: 201


                        Type ?<data object name> to get a specific information
about it, e.g., ?kegg.pathways.
                    


As the output above suggests, users can obtain help on any of the collections using the standard R help ( ?) command, for instance ?Mm.c2 will return more information on the mouse version of the c2 collection from MSigDB. The above information can be returned as a list:



                        > info = egsea.data("mouse", returnInfo = TRUE)
> names(info)
[1] "kegg"   "msigdb" "gsetdb"
> info$msigdb$info$collections
[1] "h"  "c1" "c2" "c3" "c4" "c5" "c6" "c7"

                    


To highlight the capabilities of the **EGSEA** package, the KEGG pathways, c2 (curated gene sets) and c5 (Gene Ontology gene sets) collections from the MSigDB database are selected. Next, an index is built for each gene set collection using the EGSEA indexing functions to link the genes in the different gene set collections to the rows of our RNA-seq gene expression matrix. Indexes for the c2 and c5 collections from MSigDB and for the KEGG pathways are built using the buildIdx function which relies on Entrez gene IDs as its key. In the **EGSEAdata** gene set collections, Entrez IDs are used as they are widely adopted by the different source databases and tend to be more consistent and robust since there is one identifier per gene in a gene set. It is also relatively easy to convert other gene IDs into Entrez IDs.



                        > library(EGSEA)
> gs.annots = buildIdx(entrezIDs=v$genes$ENTREZID, species="mouse",
+           msigdb.gsets=c("c2", "c5"), go.part = TRUE)
[1] "Loading MSigDB Gene Sets ... "
[1] "Loaded gene sets for the collection c2 ..."
[1] "Indexed the collection c2 ..."
[1] "Created annotation for the collection c2 ..."
[1] "Loaded gene sets for the collection c5 ..."
[1] "Indexed the collection c5 ..."
[1] "Created annotation for the collection c5 ..."
MSigDB c5 gene set collection has been partitioned into
c5BP, c5CC, c5MF
[1] "Building KEGG pathways annotation object ... "
> names(gs.annots)
[1] "c2"   "c5BP" "c5CC" "c5MF" "kegg"
                    


To obtain additional information on the gene set collection indexes, including the total number of gene sets, the version number and date of last revision, the methods *summary*, *show* and *getSetByName* (or *getSetByID*) can be invoked on an object of class **GSCollectionIndex**, which stores all of the relevant gene set information, as follows:



                        > class(gs.annots$c2)
[1] "GSCollectionIndex"
attr(,"package")
[1] "EGSEA"
> summary(gs.annots$c2)
c2 Curated Gene Sets (c2): 4726 gene sets - Version: 5.2, Update date: 07 March 2017
> show(gs.annots$c2)
An object of class "GSCollectionIndex"
Number of gene sets: 4726
Annotation columns: ID, GeneSet, BroadUrl, Description, PubMedID, NumGenes, Contributor
Total number of indexing genes: 14165
Species: Mus musculus
Collection name: c2 Curated Gene Sets
Collection unique label: c2
Database version: 5.2
Database update date: 07 March 2017
> s = getSetByName(gs.annots$c2, "SMID_BREAST_CANCER_LUMINAL_A_DN")
ID: M13072
GeneSet: SMID_BREAST_CANCER_LUMINAL_A_DN
BroadUrl: http://www.broadinstitute.org/gsea/msigdb/cards/SMID_BREAST_CANCER_LUMINAL_A_DN.html
Description: Genes down-regulated in the luminal A subtype of breast cancer.
PubMedID: 18451135
NumGenes: 23/24
Contributor: Jessica Robertson
> class(s)
[1] "list"
> names(s)
[1] "SMID_BREAST_CANCER_LUMINAL_A_DN"
> names(s$SMID_BREAST_CANCER_LUMINAL_A_DN)
[1] "ID"          "GeneSet"     "BroadUrl"     "Description" "PubMedID"
[6] "NumGenes"    "Contributor"
                    


Objects of class **GSCollectionIndex** store for each gene set the Entrez gene IDs in the slot original, the indexes in the slot idx and additional annotation for each set in the slot anno.



                        > slotNames(gs.annots$c2)
[1] "original"   "idx"       "anno"        "featureIDs" "species"
[6] "name"       "label"     "version"     "date"
                    


Other EGSEA functions such as buildCustomIdx, buildGMTIdx, buildKEGGIdx, buildMSigDBIdx and buildGeneSetDBIdx can be also used to build gene set collection indexes. The functions buildCustomIdx and buildGMTIdx were written to allow users to run EGSEA on gene set collections that may have been curated within a lab or downloaded from public databases and allow use of gene identifiers other than Entrez IDs. Example databases include, ENCODE Gene Set Hub (available from https://sourceforge.net/projects/encodegenesethub/), which is a growing resource of gene sets derived from high quality ENCODE profiling experiments encompassing hundreds of DNase hypersensitivity, histone modification and transcription factor binding experiments ^30^. Other resources include PathwayCommons ( http://www.pathwaycommons.org/) ^31^ and the **KEGGREST** ^32^ package that provides access to up-to-date KEGG pathways across many species.

### 2. Configuring EGSEA

Before an EGSEA test is carried out, a few parameters need to be specified. First, a mapping between Entrez IDs and Gene Symbols is created for use by the visualization procedures. This mapping can be extracted from the genes data.frame of the ***voom*** object as follows:



                        > colnames(v$genes)
[1] "ENTREZID" "SYMBOL"    "CHR"
> symbolsMap = v$genes[, c(1, 2)]
> colnames(symbolsMap) = c("FeatureID", "Symbols")
> symbolsMap[, "Symbols"] = as.character(symbolsMap[, "Symbols"])
                    


Another important parameter in EGSEA is the list of base GSE methods ( baseMethods in the code below), which determines the individual algorithms that are used in the ensemble testing. The supported base methods can be listed using the function egsea.base as follows:



                        > egsea.base()
 [1] "camera"     "roast"      "safe"      "gage"        "padog"      "plage"
 [7] "zscore"     "gsva"       "ssgsea"    "globaltest"  "ora"        "fry"
                    


The ***plage***, ***zscore*** and ***ssgsea*** algorithms are available in the **GSVA** package and ***camera***, ***fry*** and ***roast*** are implemented in the **limma** package ^24^. The ***ora*** method is implemented using the phyper function from the **stats** package ^33^, which estimates the hypergeometric distribution for a 2 × 2 contingency table. The remaining algorithms are implemented in Bioconductor packages of the same name. A wrapper function is provided for each individual GSE method to utilize this existing R code and create a universal interface for all methods.

Eleven base methods are selected for our EGSEA analysis: ***camera***, ***safe***, ***gage***, ***padog***, ***plage***, ***zscore***, ***gsva***, ***ssgsea***, ***globaltest***, ***ora*** and ***fry***. ***Fry*** is a fast approximation of ***roast*** that assumes equal gene-wise variances across samples to produce similar *p*-values to a roast analysis run with an infinite number of rotations, and is selected here to save time.



                        > baseMethods = egsea.base()[-2]
> baseMethods
 [1] "camera"     "safe"       "gage"       "padog"      "plage"      "zscore"
 [7] "gsva"       "ssgsea"     "globaltest" "ora"        "fry"
                    


Although, different combinations of base methods might produce different results, it has been found via simulation that including more methods gives better performance ^5^.

Since each base method generates different *p*-values, EGSEA supports six different methods from the **metap** package ^34^ for combining individual *p*-values ( *Wilkinson* ^35^ is default), which can be listed as follows:



                        > egsea.combine()
[1] "fisher"    "wilkinson" "average"  "logitp"    "sump"      "sumz"
[7] "votep"     "median"
                    


Finally, the sorting of EGSEA results plays an essential role in identifying relevant gene sets. Any of EGSEA’s combined scores or the rankings from individual base methods can be used for sorting the results.



                        > egsea.sort()
 [1] "p.value"       "p.adj"         "vote.rank"   "avg.rank"       "med.rank"
 [6] "min.pvalue"    "min.rank"      "avg.logfc"   "avg.logfc.dir"  "direction"
[11] "significance"  "camera"        "roast"       "safe"           "gage"
[16] "padog"         "plage"         "zscore"      "gsva"           "ssgsea"
[21] "globaltest"    "ora"           "fry"
                    


Although p.adj is the default option for sorting EGSEA results for convenience, we recommend the use of either med.rank or vote.rank because they efficiently utilize the rankings of individual methods and tend to produce fewer false positives ^5^.

### 3. Ensemble testing with EGSEA

Next, the EGSEA analysis is performed using the egsea function that takes a ***voom*** object, a contrasts matrix, collections of gene sets and other run parameters as follows:



                        > gsa = egsea(voom.results=v, contrasts=contr.matrix,
+         gs.annots=gs.annots, symbolsMap=symbolsMap,
+         baseGSEAs=baseMethods, sort.by="med.rank",
+         num.threads = 8, report = FALSE)
EGSEA analysis has started
##------ Fri Jun 16 09:49:11 2017 ------##
Log fold changes are estimated using limma package ...
limma DE analysis is carried out ...
Number of used cores has changed to 3
in order to avoid CPU overloading.
EGSEA is running on the provided data and c2 collection
EGSEA is running on the provided data and c5BP collection
EGSEA is running on the provided data and c5CC collection
EGSEA is running on the provided data and c5MF collection
EGSEA is running on the provided data and kegg collection
##------ Fri Jun 16 09:57:56 2017 ------##
EGSEA analysis took 525.812 seconds.
EGSEA analysis has completed
                    


In situations where the design matrix includes an intercept, a vector of integers that specify the columns of the design matrix to test using EGSEA can be passed to the contrasts argument. If this parameter is NULL, all pairwise comparisons based on v$targets$group are created, assuming that group is the primary factor in the design matrix. Likewise, all the coefficients of the primary factor are used if the design matrix has an intercept.

**EGSEA** is implemented with parallel computing features enabled using the **parallel** package ^33^ at both the method-level and experimental contrast-level. The running time of the EGSEA test depends on the base methods selected and whether report generation is enabled or not. The latter significantly increases the run time, particularly if the argument display.top is assigned a large value (> 20) and/or a large number of gene set collections are selected. EGSEA reporting functionality generates set-level plots for the top gene sets as well as collection-level plots.

The **EGSEA** package also has a function named egsea.cnt, that can perform the EGSEA test using an RNA-seq count matrix rather than a ***voom*** object, a function named egsea.ora, that can perform over-representation analysis with EGSEA reporting capabilities using only a vector of gene IDs, and the egsea.ma function that can perform EGSEA testing using a microarray expression matrix as shown later in the workflow.

***Classes used to manage the results.*** The output of the functions egsea, egsea.cnt, egsea.ora and egsea.ma is an S4 object of class **EGSEAResults**. Several S4 methods can be invoked to query this object. For example, an overview of the EGSEA analysis can be displayed using the ***show*** method as follows:



                        > show(gsa)
An object of class "EGSEAResults"
Total number of genes: 14165
Total number of samples: 9
Contrasts: BasalvsLP, BasalvsML, LPvsML
Base GSE methods: camera (limma:3.32.2), safe (safe:3.16.0), gage (gage:2.26.0), padog (PADOG:1.18.0), plage (GSVA:1.24.1), zscore (GSVA:1.24.1), gsva (GSVA:1.24.1), ssgsea (GSVA:1.24.1),
P-values combining method: wilkinson
Sorting statistic: med.rank
Organism: Mus musculus
HTML report generated: No
Tested gene set collections:
c2 Curated Gene Sets (c2): 4726 gene sets - Version: 5.2, Update date: 07 March 2017
c5 GO Gene Sets (BP) (c5BP): 4653 gene sets - Version: 5.2, Update date: 07 March 2017
c5 GO Gene Sets (CC) (c5CC): 584 gene sets - Version: 5.2, Update date: 07 March 2017
c5 GO Gene Sets (MF) (c5MF): 928 gene sets - Version: 5.2, Update date: 07 March 2017
KEGG Pathways (kegg): 287 gene sets - Version: NA, Update date: 07 March 2017
EGSEA version: 1.5.2
EGSEAdata version: 1.4.0
Use summary(object) and topSets(object, ...) to explore this object.
                    


This command displays the number of genes and samples that were included in the analysis, the experimental contrasts, base GSE methods, the method used to combine the *p*-values derived from different GSE algorithms, the sorting statistic used and the size of each gene set collection. Note that the gene set collections are identified using the labels that appear in parentheses (e.g. c2) in the output of ***show***.

### 4. Reporting EGSEA results

***Getting top ranked gene sets.*** A summary of the top 10 gene sets in each collection for each contrast in addition to the EGSEA comparative analysis can be displayed using the S4 method ***summary*** as follows:



                        > summary(gsa)
**** Top 10 gene sets in the c2 Curated Gene Sets collection ****
** Contrast BasalvsLP **
LIM_MAMMARY_STEM_CELL_DN | LIM_MAMMARY_LUMINAL_PROGENITOR_UP
MONTERO_THYROID_CANCER_POOR_SURVIVAL_UP | SMID_BREAST_CANCER_LUMINAL_A_DN
NAKAYAMA_SOFT_TISSUE_TUMORS_PCA2_UP | REACTOME_LATENT_INFECTION_OF_HOMO_SAPIENS...
REACTOME_TRANSFERRIN_ENDOCYTOSIS_AND_RECYCLING | FARMER_BREAST_CANCER_CLUSTER_2
KEGG_EPITHELIAL_CELL_SIGNALING_... | LANDIS_BREAST_CANCER_PROGRESSION_UP


                        ** Contrast BasalvsML **
LIM_MAMMARY_STEM_CELL_DN | LIM_MAMMARY_STEM_CELL_UP
LIM_MAMMARY_LUMINAL_MATURE_DN | PAPASPYRIDONOS_UNSTABLE_ATEROSCLEROTIC_PLAQUE_DN
NAKAYAMA_SOFT_TISSUE_TUMORS_PCA2_UP | LIM_MAMMARY_LUMINAL_MATURE_UP
CHARAFE_BREAST_CANCER_LUMINAL_VS_MESENCHYMAL_UP | RICKMAN_HEAD_AND_NECK_CANCER_A
YAGUE_PRETUMOR_DRUG_RESISTANCE_DN | BERTUCCI_MEDULLARY_VS_DUCTAL_BREAST_CANCER_DN


                        ** Contrast LPvsML **
LIM_MAMMARY_LUMINAL_MATURE_UP | LIM_MAMMARY_LUMINAL_MATURE_DN
PHONG_TNF_RESPONSE_VIA_P38_PARTIAL | WOTTON_RUNX_TARGETS_UP
WANG_MLL_TARGETS | PHONG_TNF_TARGETS_DN
REACTOME_PEPTIDE_LIGAND_BINDING_RECEPTORS | CHIANG_LIVER_CANCER_SUBCLASS_CTNNB1_DN
GERHOLD_RESPONSE_TO_TZD_DN | DURAND_STROMA_S_UP


                        ** Comparison analysis **
LIM_MAMMARY_LUMINAL_MATURE_DN | LIM_MAMMARY_STEM_CELL_DN
NAKAYAMA_SOFT_TISSUE_TUMORS_PCA2_UP | LIM_MAMMARY_LUMINAL_MATURE_UP
COLDREN_GEFITINIB_RESISTANCE_DN | LIM_MAMMARY_STEM_CELL_UP
CHARAFE_BREAST_CANCER_LUMINAL_VS_MESENCHYMAL_UP | LIM_MAMMARY_LUMINAL_PROGENITOR_UP
BERTUCCI_MEDULLARY_VS_DUCTAL_BREAST_CANCER_DN | MIKKELSEN_IPS_WITH_HCP_H3K27ME3


                        **** Top 10 gene sets in the c5 GO Gene Sets (BP) collection ****
** Contrast BasalvsLP **
GO_SYNAPSE_ORGANIZATION | GO_IRON_ION_TRANSPORT
GO_CALCIUM_INDEPENDENT_CELL_CELL_ADHESION_VIA_PLASMA_MEMBRANE_CELL_ADHESION_MOLECULES | GO_PH_REDUCTION
GO_HOMOPHILIC_CELL_ADHESION_VIA_PLASMA_MEMBRANE_ADHESION_MOLECULES | GO_VACUOLAR_ACIDIFICATION
GO_FERRIC_IRON_TRANSPORT | GO_TRIVALENT_INORGANIC_CATION_TRANSPORT
GO_NEURON_PROJECTION_GUIDANCE | GO_MESONEPHROS_DEVELOPMENT


                        ** Contrast BasalvsML **
GO_FERRIC_IRON_TRANSPORT | GO_TRIVALENT_INORGANIC_CATION_TRANSPORT
GO_IRON_ION_TRANSPORT | GO_NEURON_PROJECTION_GUIDANCE
GO_GLIAL_CELL_MIGRATION | GO_SPINAL_CORD_DEVELOPMENT
GO_REGULATION_OF_SYNAPSE_ORGANIZATION | GO_ACTION_POTENTIAL
GO_MESONEPHROS_DEVELOPMENT | GO_NEGATIVE_REGULATION_OF_SMOOTH_MUSCLE_CELL_MIGRATION


                        ** Contrast LPvsML **
GO_NEGATIVE_REGULATION_OF_NECROTIC_CELL_DEATH | GO_PARTURITION
GO_RESPONSE_TO_VITAMIN_D | GO_GPI_ANCHOR_METABOLIC_PROCESS
GO_REGULATION_OF_BLOOD_PRESSURE | GO_DETECTION_OF_MOLECULE_OF_BACTERIAL_ORIGIN
GO_CELL_SUBSTRATE_ADHESION | GO_PROTEIN_TRANSPORT_ALONG_MICROTUBULE
GO_INTRACILIARY_TRANSPORT | GO_CELLULAR_RESPONSE_TO_VITAMIN


                        ** Comparison analysis **
GO_IRON_ION_TRANSPORT | GO_FERRIC_IRON_TRANSPORT
GO_TRIVALENT_INORGANIC_CATION_TRANSPORT | GO_NEURON_PROJECTION_GUIDANCE
GO_MESONEPHROS_DEVELOPMENT | GO_SYNAPSE_ORGANIZATION
GO_REGULATION_OF_SYNAPSE_ORGANIZATION | GO_MEMBRANE_DEPOLARIZATION_DURING_CARDIAC_MUSCLE_CELL_ACTION_POTENTIAL
GO_HOMOPHILIC_CELL_ADHESION_VIA_PLASMA_MEMBRANE_ADHESION_MOLECULES | GO_NEGATIVE_REGULATION_OF_SMOOTH_MUSCLE_CELL_MIGRATION


                        **** Top 10 gene sets in the c5 GO Gene Sets (CC) collection ****
** Contrast BasalvsLP **
GO_PROTON_TRANSPORTING_V_TYPE_ATPASE_COMPLEX | GO_VACUOLAR_PROTON_TRANSPORTING_V_TYPE_ATPASE_COMPLEX
GO_MICROTUBULE_END | GO_MICROTUBULE_PLUS_END
GO_ACTIN_FILAMENT_BUNDLE | GO_CELL_CELL_ADHERENS_JUNCTION
GO_NEUROMUSCULAR_JUNCTION | GO_AP_TYPE_MEMBRANE_COAT_ADAPTOR_COMPLEX
GO_INTERMEDIATE_FILAMENT | GO_CONDENSED_NUCLEAR_CHROMOSOME_CENTROMERIC_REGION


                        ** Contrast BasalvsML **
GO_FILOPODIUM_MEMBRANE | GO_LATE_ENDOSOME_MEMBRANE
GO_PROTON_TRANSPORTING_V_TYPE_ATPASE_COMPLEX | GO_NEUROMUSCULAR_JUNCTION
GO_COATED_MEMBRANE | GO_ACTIN_FILAMENT_BUNDLE
GO_CLATHRIN_COAT | GO_AP_TYPE_MEMBRANE_COAT_ADAPTOR_COMPLEX
GO_CLATHRIN_ADAPTOR_COMPLEX | GO_CONTRACTILE_FIBER


                        ** Contrast LPvsML **
GO_CILIARY_TRANSITION_ZONE | GO_TCTN_B9D_COMPLEX
GO_NUCLEAR_NUCLEOSOME | GO_INTRINSIC_COMPONENT_OF_ORGANELLE_MEMBRANE
GO_ENDOPLASMIC_RETICULUM_QUALITY_CONTROL_COMPARTMENT | GO_KERATIN_FILAMENT
GO_PROTEASOME_COMPLEX | GO_CILIARY_BASAL_BODY
GO_PROTEASOME_CORE_COMPLEX | GO_CORNIFIED_ENVELOPE


                        ** Comparison analysis **
GO_PROTON_TRANSPORTING_V_TYPE_ATPASE_COMPLEX | GO_ACTIN_FILAMENT_BUNDLE
GO_NEUROMUSCULAR_JUNCTION | GO_AP_TYPE_MEMBRANE_COAT_ADAPTOR_COMPLEX
GO_CONTRACTILE_FIBER | GO_INTERMEDIATE_FILAMENT
GO_LATE_ENDOSOME_MEMBRANE | GO_CLATHRIN_VESICLE_COAT
GO_ENDOPLASMIC_RETICULUM_QUALITY_CONTROL_COMPARTMENT | GO_MICROTUBULE_END


                        **** Top 10 gene sets in the c5 GO Gene Sets (MF) collection ****
** Contrast BasalvsLP **
GO_HYDROGEN_EXPORTING_ATPASE_ACTIVITY | GO_SIGNALING_PATTERN_RECOGNITION_RECEPTOR_ACTIVITY
GO_LIPID_TRANSPORTER_ACTIVITY | GO_TRIGLYCERIDE_LIPASE_ACTIVITY
GO_AMINE_BINDING | GO_STRUCTURAL_CONSTITUENT_OF_MUSCLE
GO_NEUROPEPTIDE_RECEPTOR_ACTIVITY | GO_WIDE_PORE_CHANNEL_ACTIVITY
GO_CATION_TRANSPORTING_ATPASE_ACTIVITY | GO_LIPASE_ACTIVITY


                        ** Contrast BasalvsML **
GO_G_PROTEIN_COUPLED_RECEPTOR_ACTIVITY | GO_TRANSMEMBRANE_RECEPTOR_PROTEIN_KINASE_ACTIVITY
GO_STRUCTURAL_CONSTITUENT_OF_MUSCLE | GO_VOLTAGE_GATED_SODIUM_CHANNEL_ACTIVITY
GO_CORECEPTOR_ACTIVITY | GO_TRANSMEMBRANE_RECEPTOR_PROTEIN_TYROSINE_KINASE_ACTIVITY
GO_LIPID_TRANSPORTER_ACTIVITY | GO_SULFOTRANSFERASE_ACTIVITY
GO_CATION_TRANSPORTING_ATPASE_ACTIVITY | GO_PEPTIDE_RECEPTOR_ACTIVITY


                        ** Contrast LPvsML **
GO_MANNOSE_BINDING | GO_PHOSPHORIC_DIESTER_HYDROLASE_ACTIVITY
GO_BETA_1_3_GALACTOSYLTRANSFERASE_ACTIVITY | GO_COMPLEMENT_BINDING
GO_ALDEHYDE_DEHYDROGENASE_NAD_ACTIVITY | GO_MANNOSIDASE_ACTIVITY
GO_LIGASE_ACTIVITY_FORMING_CARBON_NITROGEN_BONDS | GO_CARBOHYDRATE_PHOSPHATASE_ACTIVITY
GO_LIPASE_ACTIVITY | GO_PEPTIDE_RECEPTOR_ACTIVITY


                        ** Comparison analysis **
GO_STRUCTURAL_CONSTITUENT_OF_MUSCLE | GO_LIPID_TRANSPORTER_ACTIVITY
GO_CATION_TRANSPORTING_ATPASE_ACTIVITY | GO_CHEMOREPELLENT_ACTIVITY
GO_HEPARAN_SULFATE_PROTEOGLYCAN_BINDING | GO_TRANSMEMBRANE_RECEPTOR_PROTEIN_TYROSINE_KINASE_ACTIVITY
GO_LIPASE_ACTIVITY | GO_PEPTIDE_RECEPTOR_ACTIVITY
GO_CORECEPTOR_ACTIVITY | GO_TRANSMEMBRANE_RECEPTOR_PROTEIN_KINASE_ACTIVITY


                        **** Top 10 gene sets in the KEGG Pathways collection ****
** Contrast BasalvsLP **
Collecting duct acid secretion | alpha-Linolenic acid metabolism
Synaptic vesicle cycle | Hepatitis C
Vascular smooth muscle contraction | Rheumatoid arthritis
cGMP-PKG signaling pathway | Axon guidance
Progesterone-mediated oocyte maturation | Arrhythmogenic right ventricular cardiomyopathy (ARVC)


                        ** Contrast BasalvsML **
Collecting duct acid secretion | Synaptic vesicle cycle
Other glycan degradation | Axon guidance
Arrhythmogenic right ventricular cardiomyopathy (ARVC) | Glycerophospholipid metabolism
Lysosome | Vascular smooth muscle contraction
Protein digestion and absorption | Oxytocin signaling pathway


                        ** Contrast LPvsML **
Glycosylphosphatidylinositol(GPI)-anchor biosynthesis | Histidine metabolism
Drug metabolism - cytochrome P450 | PI3K-Akt signaling pathway
Proteasome | Sulfur metabolism
Renin-angiotensin system | Nitrogen metabolism
Tyrosine metabolism | Systemic lupus erythematosus


                        ** Comparison analysis **
Collecting duct acid secretion | Synaptic vesicle cycle
Vascular smooth muscle contraction | Axon guidance
Arrhythmogenic right ventricular cardiomyopathy (ARVC) | Oxytocin signaling pathway
Lysosome | Adrenergic signaling in cardiomyocytes
Linoleic acid metabolism | cGMP-PKG signaling pathway
                    


EGSEA’s *comparative* analysis allows researchers to estimate the significance of a gene set across multiple experimental contrasts. This analysis helps in the identification of biological processes that are perturbed in multiple experimental conditions simultaneously. This experiment is the RNA-seq equivalent of Lim *et al.* (2010) ^22^, who used Illumina microarrays to study the same cell populations (see later), so it is reassuring to observe the LIM gene signatures derived from this experiment amongst the top ranked c2 gene signatures in both the individual contrasts and comparative results.

Another way of exploring the EGSEA results is to retrieve the top ranked *N* sets in each collection and contrast using the method ***topSets***. For example, the top 10 gene sets in the c2 collection for the comparative analysis can be retrieved as follows:



                        > topSets(gsa, gs.label="c2", contrast = "comparison", names.only=TRUE)

                        Extracting the top gene sets of the collection

                        c2 Curated Gene Sets for the contrast comparison
 
                        Sorted by med.rank
 
                        [1] "LIM_MAMMARY_LUMINAL_MATURE_DN"
 
                        [2] "LIM_MAMMARY_STEM_CELL_DN"
 
                        [3] "NAKAYAMA_SOFT_TISSUE_TUMORS_PCA2_UP"
 
                        [4] "LIM_MAMMARY_LUMINAL_MATURE_UP"
 
                        [5] "COLDREN_GEFITINIB_RESISTANCE_DN"
 
                        [6] "LIM_MAMMARY_STEM_CELL_UP"
 
                        [7] "CHARAFE_BREAST_CANCER_LUMINAL_VS_MESENCHYMAL_UP"
 
                        [8] "LIM_MAMMARY_LUMINAL_PROGENITOR_UP"
 
                        [9] "BERTUCCI_MEDULLARY_VS_DUCTAL_BREAST_CANCER_DN"

                        [10] "MIKKELSEN_IPS_WITH_HCP_H3K27ME3"
                    


The gene sets are ordered based on their med.rank as selected when ***egsea*** was invoked above. When the argument names.only is set to FALSE, additional information is displayed for each gene set including gene set annotation, the EGSEA scores and the individual rankings by each base method. As expected, gene sets retrieved by EGSEA included the LIM gene sets ^22^ that were derived from microarray profiles of analagous mammary cell populations (sets 1, 2, 4, 6 and 8) as well as those derived from populations with similar origin (sets 7 and 9) and behaviour or characteristics (sets 5 and 10).

Next, ***topSets*** can be used to search for gene sets of interest based on different EGSEA scores as well as the rankings of individual methods. For example, the ranking of the six LIM gene sets from the c2 collection can be displayed based on the med.rank as follows:



                        > t = topSets(gsa, contrast = "comparison",
+             names.only=FALSE, number = Inf, verbose = FALSE)
> t[grep("LIM_", rownames(t)), c("p.adj", "Rank", "med.rank", "vote.rank")]
                                          p.adj Rank med.rank vote.rank
LIM_MAMMARY_LUMINAL_MATURE_DN      1.646053e-29    1       36         5
LIM_MAMMARY_STEM_CELL_DN           6.082053e-43    2       37         5
LIM_MAMMARY_LUMINAL_MATURE_UP      2.469061e-22    4       92         5
LIM_MAMMARY_STEM_CELL_UP          3.154132e-103    6      134         5
LIM_MAMMARY_LUMINAL_PROGENITOR_UP  3.871536e-30    8      180         5
LIM_MAMMARY_LUMINAL_PROGENITOR_DN  2.033005e-06  178      636       115
                    


While five of the LIM gene sets are ranked in the top 10 by EGSEA, the values shown in the median rank ( med.rank) column indicate that individual methods can assign much lower ranks to these sets. EGSEA’s prioritisation of these gene sets demonstrates the benefit of an ensemble approach.

Similarly, we can find the top 10 pathways in the KEGG collection from the ensemble analysis for the Basal versus LP contrast and the comparative analysis as follows:



                        > topSets(gsa, gs.label="kegg", contrast="BasalvsLP", sort.by="med.rank")

                        Extracting the top gene sets of the collection

                        KEGG Pathways for the contrast BasalvsLP
 
                        Sorted by med.rank
 
                        [1] "Collecting duct acid secretion"            "alpha-Linolenic acid metabolism"
 
                        [3] "Synaptic vesicle cycle"                    "Hepatitis C"
 
                        [5] "Vascular smooth muscle contraction"        "Rheumatoid arthritis"
 
                        [7] "cGMP-PKG signaling pathway"                "Axon guidance"
 
                        [9] "Progesterone-mediated oocyte maturation"   "Arrhythmogenic right ventricular cardiomyopathy (ARVC)"
 

                        > topSets(gsa, gs.label="kegg", contrast="comparison", sort.by="med.rank")

                        Extracting the top gene sets of the collection

                        KEGG Pathways for the contrast comparison
 
                        Sorted by med.rank
 
                        [1] "Collecting duct acid secretion"            "Synaptic vesicle cycle"
 
                        [3] "Vascular smooth muscle contraction"        "Axon guidance"
 
                        [5] "Arrhythmogenic right ventricular cardiomyopathy (ARVC)"  "Oxytocin signaling pathway"
 
                        [7] "Lysosome"                                  "Adrenergic signaling in cardiomyocytes"
 
                        [9] "Linoleic acid metabolism"                  "cGMP-PKG signaling pathway"
                    


EGSEA highlights many pathways with known importance in the mammary gland such as those associated with distinct roles in lactation like basal cell contraction ( Vascular smooth muscle contraction and Oxytocin signalling pathway) and milk production and secretion from luminal lineage cells ( Collecting duct acid secretion, Synaptic vesicle cycle and Lysosome).

**Figure 2.**
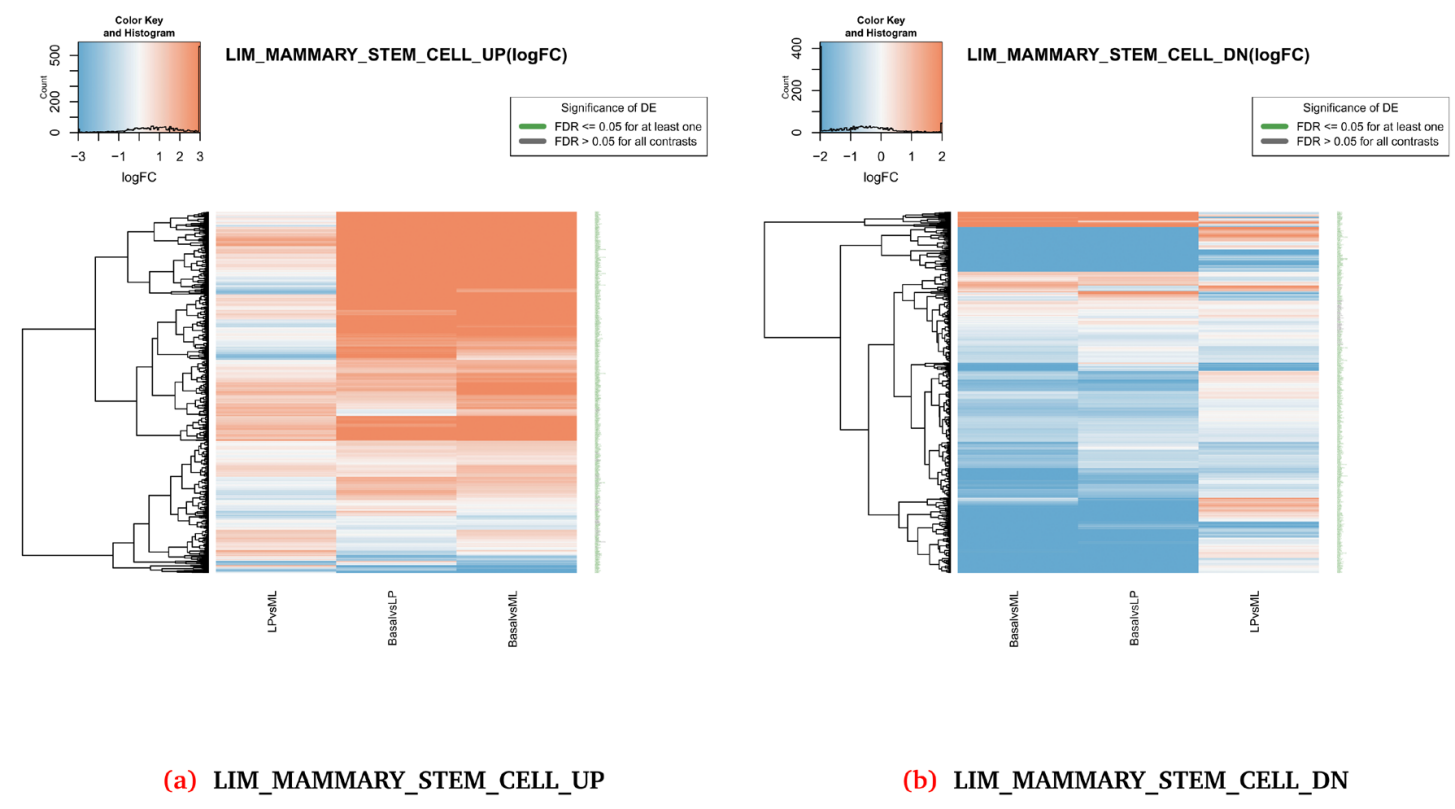
Heatmaps of log-fold-changes for genes in the *LIM_MAMMARY_STEM_CELL_UP* and *LIM_MAMMARY_STEM_CELL_DN* gene sets across the three experimental comparisons (Basal vs LP, Basal vs ML and LP vs ML).



***Visualizing results at the gene set level.*** Graphical representation of gene expression patterns within and between gene sets is an essential part of communicating the results of an analysis to collaborators and other researchers. **EGSEA** enables users to explore the elements of a gene set via a heatmap using the ***plotHeatmap*** method. Figure 2 shows examples for the LIM_MAMMARY_STEM_CELL_UP and LIM_MAMMARY_STEM_CELL_DN signatures which can be visualized across all contrasts using the code below.

                        > plotHeatmap(gsa, gene.set="LIM_MAMMARY_STEM_CELL_UP", gs.label="c2",
+         contrast = "comparison", file.name = "hm_cmp_LIM_MAMMARY_STEM_CELL_UP")
Generating heatmap for LIM_MAMMARY_STEM_CELL_UP from the collection
c2 Curated Gene Sets and for the contrast comparison
> plotHeatmap(gsa, gene.set="LIM_MAMMARY_STEM_CELL_DN", gs.label="c2",
+         contrast = "comparison", file.name = "hm_cmp_LIM_MAMMARY_STEM_CELL_DN")
Generating heatmap for LIM_MAMMARY_STEM_CELL_DN from the collection
c2 Curated Gene Sets and for the contrast comparison
                    


When using ***plotHeatmap***, the gene.set value must match the name returned from the ***topSets*** method. The rows of the heatmap represent the genes in the set and the columns represent the experimental contrasts. The heatmap colour-scale ranges from down-regulated (blue) to up-regulated (red) while the row labels (Gene symbols) are coloured in green when the genes are statistically significant in the DE analysis (i.e. FDR *≤* 0.05 in at least one contrast). Heatmaps can be generated for individual comparisons by changing the contrast argument of ***plotHeatmap***. The ***plotHeatmap*** method also generates a CSV file that includes the DE analysis results from ***limma::topTable*** for all expressed genes in the selected gene set and for each contrast (in the case of contrast = "comparison"). This file can be used to create customised plots using other R/Bioconductor packages.

In addition to heatmaps, pathway maps can be generated for the KEGG gene sets using the ***plotPathway*** method which uses functionality from the **pathview** package ^36^. For example, the third KEGG signalling pathway retrieved for the contrast BasalvsLP is Vascular smooth muscle contraction and can be visualized as follows:



                        > plotPathway(gsa, gene.set = "Vascular smooth muscle contraction",
+             contrast = "BasalvsLP", gs.label = "kegg",
+             file.name = "Vascular_smooth_muscle_contraction")
Generating pathway map for Vascular smooth muscle contraction from the collection
KEGG Pathways and for the contrast BasalvsLP
                    


**Figure 3.**
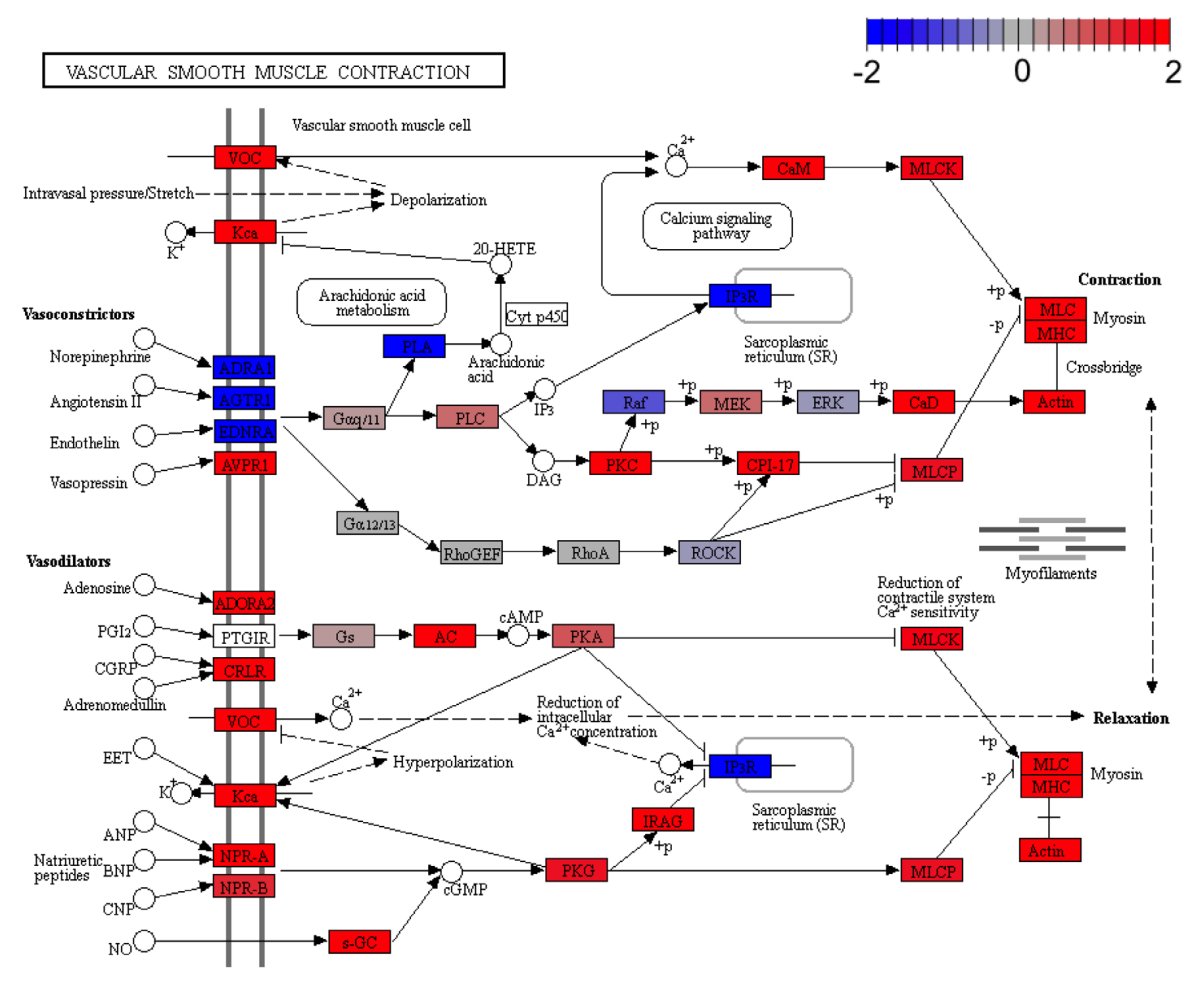
Pathway map for Vascular smooth muscle contraction (KEGG pathway mmu04270) with log-fold-changes from the Basal vs LP contrast.



Pathway components are coloured based on the gene-specific log-fold-changes as calculated in the **limma** DE analysis ( Figure 3). Similarly, a comparative map can be generated for a given pathway across all contrasts.

                        > plotPathway(gsa, gene.set = "Vascular smooth muscle contraction",
+             contrast = "comparison", gs.label = "kegg",
+             file.name = "Vascular_smooth_muscle_contraction_cmp")
Generating pathway map for Vascular smooth muscle contraction from the collection
KEGG Pathways and for the contrast comparison
                    


**Figure 4.**
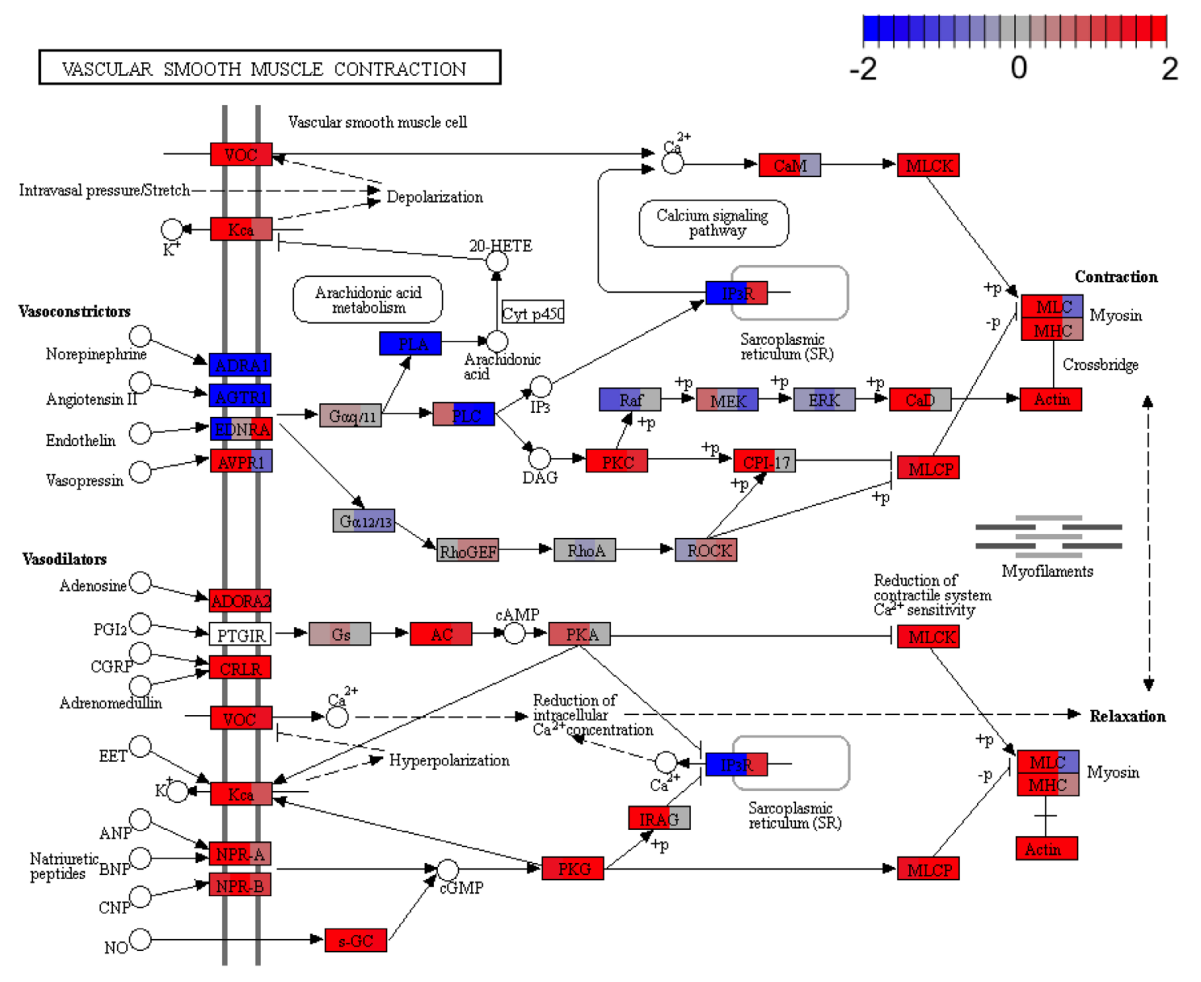
Pathway map for Vascular smooth muscle contraction (KEGG pathway mmu04270) with log-fold-changes across three experimental contrasts shown for each gene in the same order left to right that they appear in the contrasts matrix (i.e. Basal vs LP, Basal vs ML and LP vs ML).

The comparative pathway map shows the log-fold-changes for each gene in each contrast by dividing the gene nodes on the map into multiple columns, one for each contrast ( Figure 4).

**Figure 5.**
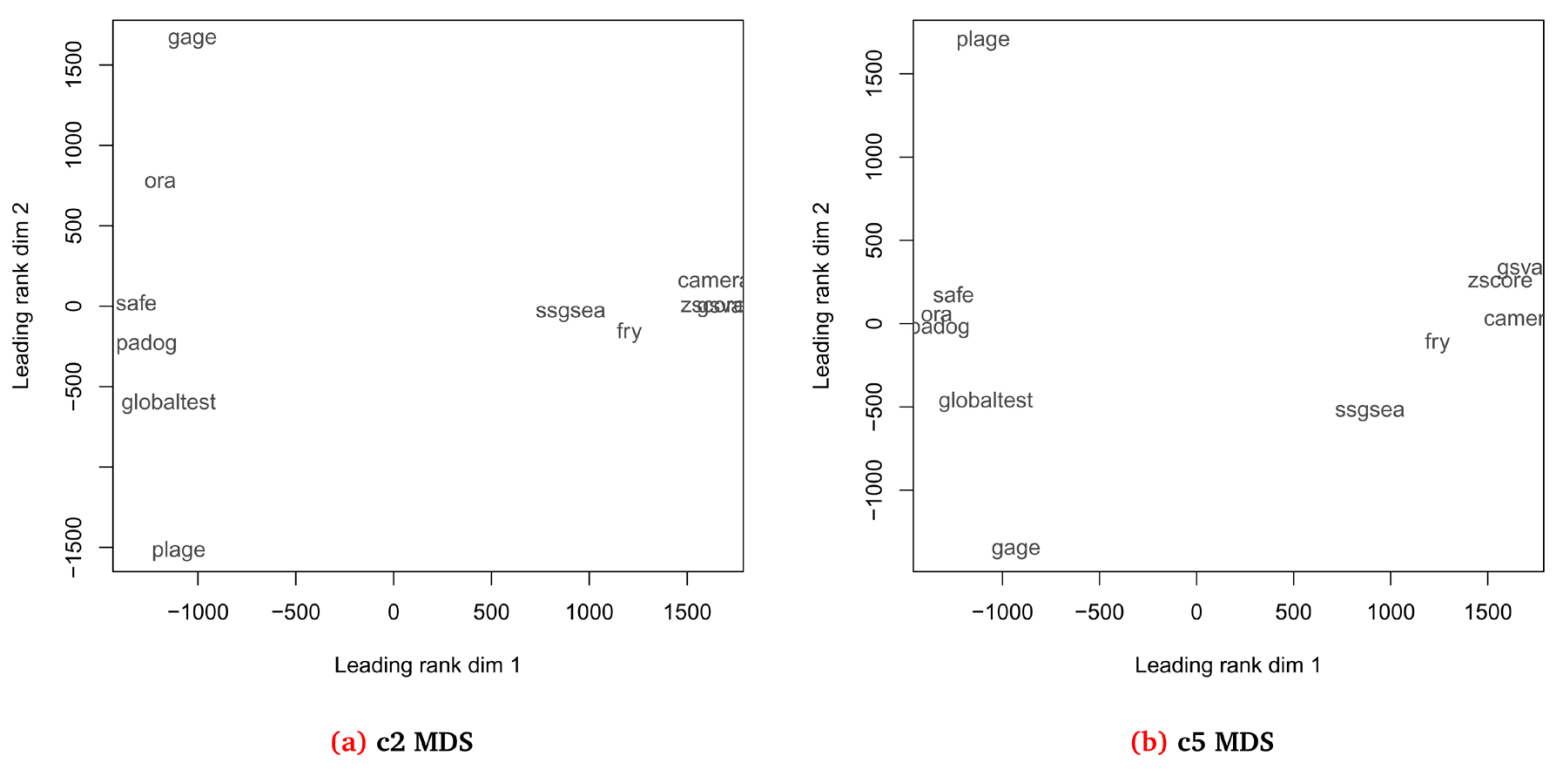
Multi-dimensional scaling (MDS) plot showing the relationship between different gene set testing methods based on the rankings of the c2 ( **a**) and c5 ( **b**) gene sets on the Basal vs LP contrast.

***Visualizing results at the experiment level.*** Since **EGSEA** combines the results from multiple gene set testing methods, it can be interesting to compare how different base methods rank a given gene set collection for a selected contrast. The plotMethods command generates a multi-dimensional scaling (MDS) plot for the ranking of gene sets across all the base methods used ( Figure 5). Methods that rank gene sets similarly will appear closer together in this plot and we see that certain methods consistently cluster together across different gene set collections. The clustering of methods does not necessarily follow the style of null hypothesis tested though (i.e. *self-contained* versus *competitive*).



                        > plotMethods(gsa, gs.label = "c2", contrast = "BasalvsLP",
+         file.name = "mds_c2_BasalvsLP")
Generating MDS plot for the collection
c2 Curated Gene Sets and for the contrast BasalvsLP
> plotMethods(gsa, gs.label = "c5BP", contrast = "BasalvsLP",
+         file.name = "mds_c5_BasalvsLP")
Generating MDS plot for the collection
c5BP GO Gene Sets and for the contrast BasalvsLP
                    


The significance of each gene set in a given collection for a selected contrast can be visualized using EGSEA’s plotSummary method.



                        > plotSummary(gsa, gs.label = 3, contrast = 3,
+         file.name = "summary_kegg_LPvsML")
Generating Summary plots for the collection
KEGG Pathways and for the contrast LPvsML
                    


**Figure 6.**
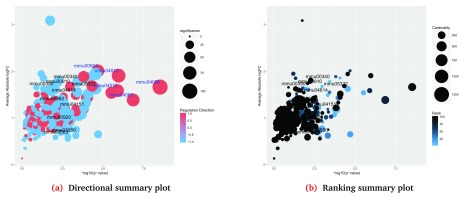
Summary plots of the significance of all gene sets in the KEGG collection for the LP vs ML contrast.

The summary plot visualizes the gene sets as bubbles based on the − log _10_ ( *p*- *value*) (X-axis) and the average absolute log fold-change of the set genes (Y-axis). The sets that appear towards the top-right corner of this plot are most likely to be biologically relevant. EGSEA generates two types of summary plots: the directional summary plot ( Figure 6a), which colours the bubbles based on the regulation direction of the gene set (the direction of the majority of genes), and the ranking summary plot ( Figure 6b), which colours the bubbles based on the gene set ranking in a given collection (according to the sort.by argument). The bubble size is based on the EGSEA *significance score* in the former plot and the gene set size in the latter. For example, the summary plots of the KEGG pathways for the LP vs ML contrast show few significant pathways ( Figure 6). The blue colour labels on the ranking plot represents gene sets that do not appear in the top 10 gene sets that are selected based on the sort.by argument, yet their EGSEA *significance scores* are among the top 5 in the entire collection based on the *significance score*. This is used to identify gene sets with high *significance scores* that were not captured by the sort.by score. The gene set IDs and more information about each set can be found in the EGSEA HTML report generated later.

**Figure 7.**
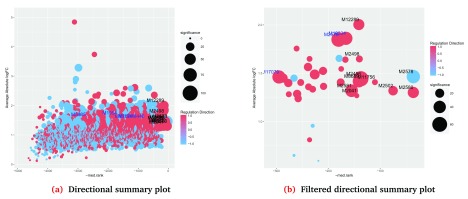
Summary plots of the significance of selected gene sets in the c2 collection for the LP vs ML contrast. The x-axis in each plot is the med.rank. A cut-off of 300 was used to select significant gene sets in the filtered plot ( **b**).

By default, ***plotSummary*** uses a gene set’s p.adj score for the X-axis. This behaviour can be easily modified by assigning any of the available ***sort.by*** scores into the parameter x.axis, for example, med.rank can be used to create an EGSEA summary plot ( Figure 7a) as follows:



                        > plotSummary(gsa, gs.label = 1, contrast = 3,
+         file.name = "summary_c2_LPvsML",
+         x.axis = "med.rank")
Generating Summary plots for the collection
c2 Curated Gene Sets and for the contrast LPvsML
                    


The summary plot tends to become cluttered when the size of the gene set collection is very large as in Figure 7a. The parameter x.cutoff can be used to focus in on the significant gene sets rather than plotting the entire gene set collection, for example ( Figure 7b):



                        > plotSummary(gsa, gs.label = 1, contrast = 3,
+         file.name = "summary_sig_c2_LPvsML",
+         x.axis = "med.rank", x.cutoff=300)
Generating Summary plots for the collection
c2 Curated Gene Sets and for the contrast LPvsML
                    


**Figure 8.**
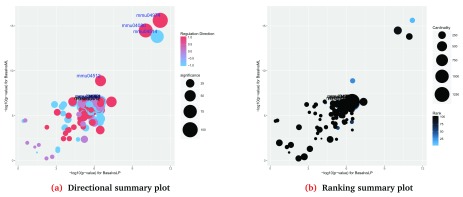
Comparative summary plots of the significance of all gene sets in the KEGG collection for the comparison of the contrasts: Basal vs LP and Basal vs ML.

Comparative summary plots can be also generated to compare the significance of gene sets between two contrasts, for example, the comparison between Basal vs LP and Basal vs ML ( Figure 8a) shows that most of the KEGG pathways are regulated in the same direction with relatively few pathways regulated in opposite directions (purple coloured bubbles in Figure 8a). Such figures can be generated using the *plotSummary* method as follows:



                        > plotSummary(gsa, gs.label = "kegg", contrast = c(1,2),
+         file.name = "summary_kegg_1vs2")
Generating Summary plots for the collection
KEGG Pathways and for the comparison BasalvsLP vs BasalvsML
                    


The ***plotSummary*** method has two useful parameters: (i) use.names that can be used to display gene set names instead of gene set IDs and (ii) interactive that can be used to generate an interactive version of this plot.

**Figure 9.**
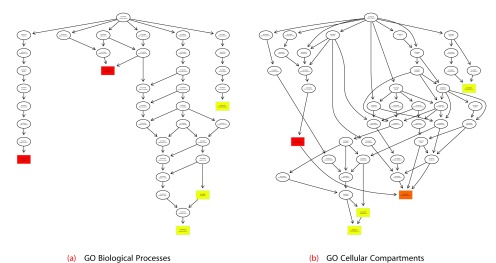
GO graphs of the top significant GO terms from the c5 gene set collection for the contrast Basal vs LP.

The c5 collection of MSigDB and the Gene Ontology collection of GeneSetDB contain Gene Ontology (GO) terms. These collections are meant to be non-redundant, containing only a small subset of the entire GO and visualizing how these terms are related to each other can be informative. **EGSEA** utilizes functionality from the **topGO** package ^37^ to generate GO graphs for the significant biological processes (BPs), cellular compartments (CCs) and molecular functions (MFs). The ***plotGOGraph*** method can generate such a display ( Figure 9) as follows:



                        > plotGOGraph(gsa, gs.label="c5BP", contrast = 1, file.name="BasalvsLP-c5BP-top-")
Generating GO Graphs for the collection c5 GO Gene Sets (BP)
 and for the contrast BasalvsLP based on the med.rank
> plotGOGraph(gsa, gs.label="c5CC", contrast = 1, file.name="BasalvsLP-c5CC-top-")
Generating GO Graphs for the collection c5 GO Gene Sets (CC)
 and for the contrast BasalvsLP based on the med.rank
                    


The GO graphs are coloured based on the values of the argument sort.by, which in this instance was taken as med.rank by default since this was selected when EGSEA was invoked. The top five most significant GO terms are highlighted by default in each GO category (MF, CC or BP). More terms can be displayed by changing the value of the parameter noSig. However, this might generate very complicated and unresolved graphs. The colour of the nodes varies between red (most significant) and yellow (least significant). The values of the sort.by scoring function are scaled between 0 and 1 to generate these graphs.

**Figure 10.**
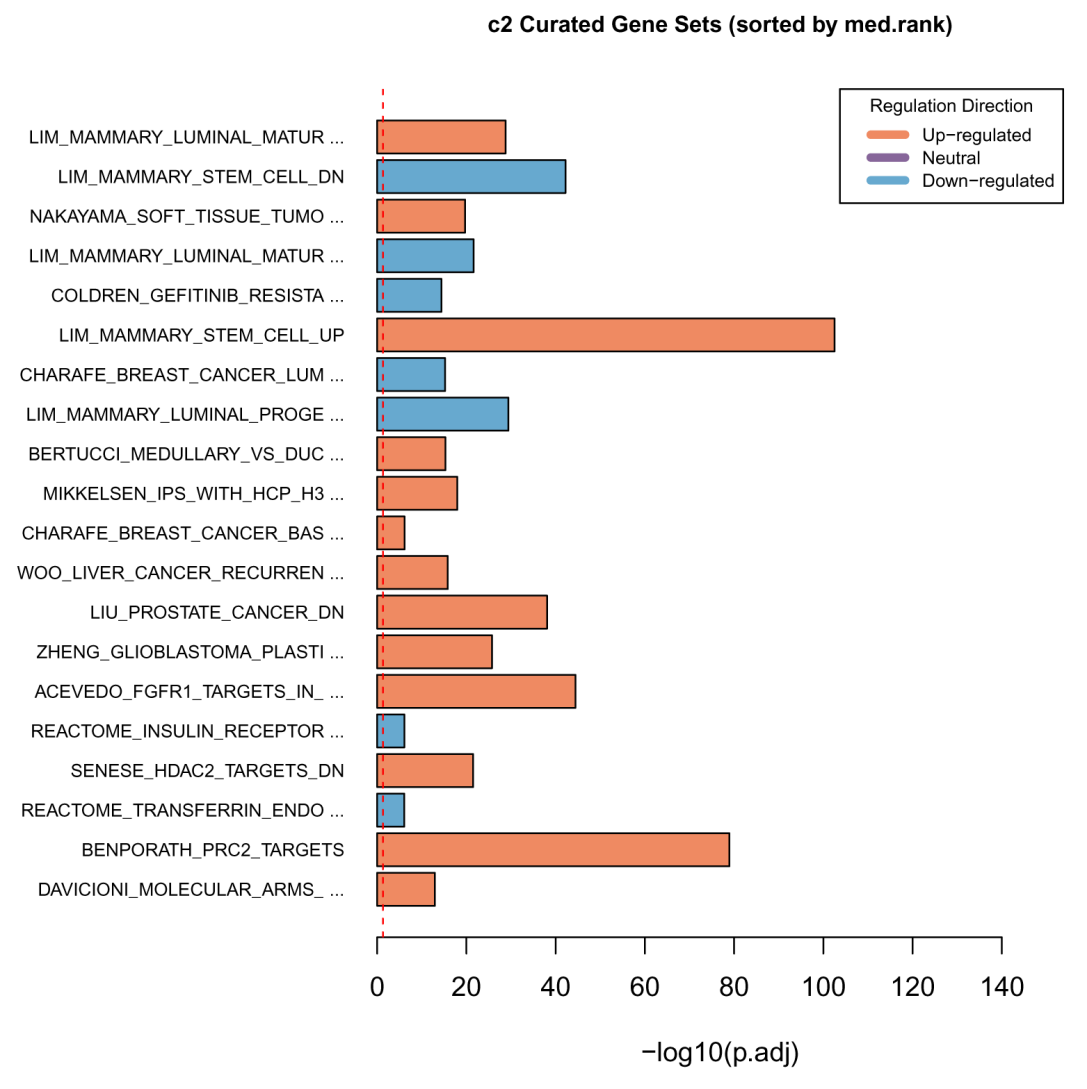
Bar plot of the *-log10(p-value)* of the top 20 gene sets from the comparative analysis of the c2 collection.

Another way to visualize results at the experiment level is via a summary *bar plot*. The method ***plotBars*** can be used to generate a bar plot for the top N gene sets in an individual collection for a particular contrast or from a comparative analysis across multiple contrasts. For example, the top 20 gene sets of the comparative analysis carried out on the c2 collection of MSigDB can be visualized in a *bar plot* ( Figure 10) as follows:



                        > plotBars(gsa, gs.label = "c2", contrast = "comparison", file.name="comparison-c2-bars")
Generating a bar plot for the collection c2 Curated Gene Sets
 and the contrast comparison
                    


The colour of the bars is based on the regulation direction of the gene sets, i.e., red for up-regulated, blue for down-regulated and purple for neutral regulation (in the case of the comparative analysis on experimental contrasts that show opposite behaviours). By default, the − log _10_( *p*. *ad j*) values are plotted for the top 20 gene sets selected and ordered based on the sort.by parameter. The parameters bar.vals, number and sort.by of ***plotBars*** can be changed to customize the *bar plot*.

**Figure 11.**
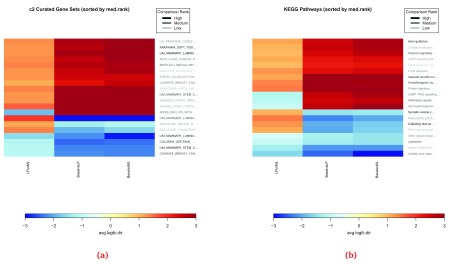
Summary heatmaps for the top 20 gene sets from the c2 ( **a**) and KEGG ( **b**) collections obtained from the EGSEA comparative analysis.

When changes over multiple conditions are of interest, a *summary heatmap* can be a useful visualization. The method ***plotSummaryHeatmaps*** generates a heatmap of the top N gene sets in the comparative analysis across all experimental conditions ( Figure 11). By default, 20 gene sets are selected based on the sort.by parameter and the values plotted are the average log-fold changes at the set level for the genes regulated in the same direction as the set regulation direction, i.e. avg.logfc.dir. The parameters number, sort.by and hm.vals of the ***plotSummaryHeatmaps*** can be used to customize the summary heatmap. Additionally, the parameter show.vals can be used to display the values of a specific EGSEA score on the heatmap cells. An example summary heatmap can be generated for the MSigDB c2 collection with the following code:



                        > plotSummaryHeatmap(gsa, gs.label="c2", hm.vals = "avg.logfc.dir",
+         file.name="summary_heatmaps_c2")
Generating summary heatmap for the collection c2 Curated Gene Sets
sort.by: med.rank, hm.vals: avg.logfc.dir, show.vals:
> plotSummaryHeatmap(gsa, gs.label="kegg", hm.vals = "avg.logfc.dir",
+         file.name="summary_heatmaps_kegg")
Generating summary heatmap for the collection KEGG Pathways
sort.by: med.rank, hm.vals: avg.logfc.dir, show.vals:
                    


We find the heatmap view at both the gene set and summary level and the summary level bar plots to be useful summaries to include in publications to highlight the gene set testing results. The top differentially expressed genes from each contrast can be accessed from the **EGSEAResults** object using the ***limmaTopTable*** method.



                        > t = limmaTopTable(gsa, contrast=1)
> head(t)
       ENTREZID   SYMBOL CHR logFC AveExpr     t  P.Value adj.P.Val    B
19253     19253   Ptpn18   1 -5.63    4.13 -34.5 5.87e-10  9.62e-07 13.2
16324     16324    Inhbb   1 -4.79    6.46 -33.2 7.99e-10  9.62e-07 13.3
53624     53624    Cldn7  11 -5.51    6.30 -40.2 1.75e-10  9.62e-07 14.5
218518   218518 Marveld2  13 -5.14    4.93 -34.8 5.56e-10  9.62e-07 13.5
12759     12759      Clu  14 -5.44    8.86 -41.0 1.52e-10  9.62e-07 14.7
70350     70350    Basp1  15 -6.07    5.25 -34.3 6.22e-10  9.62e-07 13.3
                    


***Creating an HTML report of the results.*** To generate an EGSEA HTML report for this dataset, you can either set report=TRUE when you invoke ***egsea*** or use the S4 method ***generateReport*** as follows:



                        > generateReport(gsa, number = 20, report.dir="./mam-rnaseq-egsea-report")
EGSEA HTML report is being generated ...
                    


**Figure 12.**
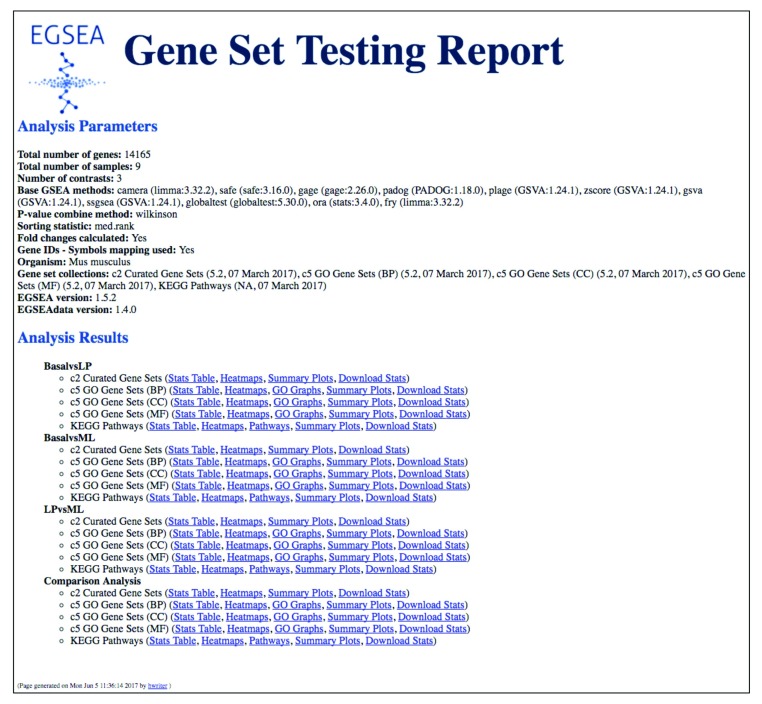
The EGSEA HTML report main page. This summary page details the analysis parameters (methods combined and ranking options selected) and organises the gene set analysis results by contrast, with further separation by gene set collection. The final section on this page presents results from the comparative analysis. For each contrast and gene set collection analysed, links to tables of results and plots are provided.

The EGSEA report generated for this dataset is available online at http://bioinf.wehi.edu.au/EGSEA/mam-rnaseq-egsea-report/index.html ( Figure 12). The HTML report is a convenient means of organising all of the results generated up to now, from the individual tables to the gene set level heatmaps, pathway maps and summary level plots. It can easily be shared with collaborators to allow them to explore their results more fully. Interactive tables of results via the **DT** package ( https://CRAN.R-project.org/package=DT) and summary plots from plotly ( https://CRAN.R-project.org/package=plotly) are integrated into the report using **htmlwidgets** ( https://CRAN.R-project.org/package=htmlwidgets) and can be added by setting interactive = TRUE in the command above. This option significantly increases both the run time and size of the final report due to the large number of gene sets in most collections.

This example completes our overview of EGSEA’s gene set testing and plotting capabilities for RNA-seq data. Readers can refer to the EGSEA vignette or individual help pages for further details on each of the above methods and classes.

## Analysis of microarray data with EGSEA

The second dataset analysed in this workflow comes from Lim *et al.* (2010) ^22^ and is the microarray equivalent of the RNA-seq data analysed above. Support for microarray data is a new feature in EGSEA, and in this example, we show an express route for analysis according to the steps shown in Figure 1, from selecting gene sets and building indexes, to configuring EGSEA, testing and reporting the results. First, the data must be appropriately preprocessed for an EGSEA analysis and to do this we make use of functions available in limma.

## Reading, preprocessing and normalisation of microarray data

To analyse this dataset, we begin by unzipping the files downloaded from http://bioinf.wehi.edu.au/EGSEA/arraydata.zip into the current working directory. Illumina BeadArray data can be read in directly using the readIDAT and readBGX functions from the illuminaio package ^38^. However, a more convenient way is via the read.idat function in **limma** which uses these **illuminaio** functions and outputs the data as an **EListRaw** object for further processing.



                    > library(limma)
> targets = read.delim("targets.txt", header=TRUE, sep=" ")
> data = read.idat(as.character(targets$File),
+                   bgxfile="GPL6887_MouseWG-6_V2_0_R0_11278593_A.bgx",
+                   annotation=c("Entrez_Gene_ID","Symbol", "Chromosome"))
Reading manifest file GPL6887_MouseWG-6_V2_0_R0_11278593_A.bgx ... Done
 4481850214_B_Grn.idat ... Done
 4481850214_C_Grn.idat ... Done
 4481850214_D_Grn.idat ... Done
 4481850214_F_Grn.idat ... Done
 4481850187_A_Grn.idat ... Done
 4481850187_B_Grn.idat ... Done
 4481850187_D_Grn.idat ... Done
 4481850187_E_Grn.idat ... Done
 4481850187_F_Grn.idat ... Done
 4466975058_A_Grn.idat ... Done
 4466975058_B_Grn.idat ... Done
 4466975058_C_Grn.idat ... Done
 4466975058_D_Grn.idat ... Done
 4466975058_E_Grn.idat ... Done
 4466975058_F_Grn.idat ... Done
Finished reading data.
> data$other$Detection = detectionPValues(data)
> data$targets = targets
> colnames(data) = targets$Sample
                


Next the neqc function in **limma** is used to carry out *normexp* background correction and quantile normalisation on the raw intensity values using negative control probes ^39^. This is followed by log _2_-transformation of the normalised intensity values and removal of the control probes.



                    > data = neqc(data)
                


We then filter out probes that are consistently non-expressed or lowly expressed throughout all samples as they are uninformative in downstream analysis. Our threshold for expression requires probes to have a detection *p*-value of less than 0.05 in at least 5 samples (the number of samples within each group). We next remove genes without a valid Entrez ID and in cases where there are multiple probes targeting different isoforms of the same gene, select the probe with highest average expression as the representative one to use in the EGSEA analysis. This leaves 7,123 probes for further analysis.



                    > table(targets$Celltype)
Basal    LP    ML
    5     5     5
> keep.exprs = rowSums(data$other$Detection<0.05)>=5
> table(keep.exprs)
keep.exprs
FALSE  TRUE
23638 21643
> data = data[keep.exprs,]
> dim(data)
[1] 21643    15
> head(data$genes)
      Probe_Id Array_Address_Id Entrez_Gene_ID        Symbol Chromosome
3 ILMN_1219601          2030280           <NA> C920011N12Rik
4 ILMN_1252621          1980164         101142 2700050P07Rik          6
6 ILMN_3162407          6220026           <NA>         Zfp36
7 ILMN_2514723          2030072           <NA> 1110067B18Rik
8 ILMN_2692952          6040743         329831 4833436C18Rik          4
9 ILMN_1257952          7160091           <NA> B930060K05Rik
> sum(is.na(data$genes$Entrez_Gene_ID))
[1] 11535
> data1 = data[!is.na(data$genes$Entrez_Gene_ID), ]
> dim(data1)
[1] 10108    15
> ord = order(lmFit(data1)$Amean, decreasing=TRUE)
> ids2keep = data1$genes$Array_Address_Id[ord][!duplicated(data1$genes$Entrez_Gene_ID[ord])]
> data1 = data1[match(ids2keep, data1$genes$Array_Address_Id),]
> dim(data1)
[1] 7123   15
> expr = data1$E
> group = as.factor(data1$targets$Celltype)
> probe.annot = data1$genes[, 2:4]
> head(probe.annot)
> head(probe.annot)
      Array_Address_Id Entrez_Gene_ID   Symbol
39513          4120224          20102    Rps4x
9062           2260576          22143   Tuba1b
15308          5720202          12192  Zfp36l1
39894          1470600          11947    Atp5b
24709          2710477          20088    Rps24
9872           1580471         228033   Atp5g3
                


## Setting up the linear model for EGSEA testing

As before, we need to set up an appropriate linear model ^29^ and contrasts matrix to look for differences between the Basal and LP, Basal and ML and LP and ML populations. A batch term is included in the linear model to account for differences in expression that are attributable to the day the experiment was run.



                    > head(data1$targets)
                     File Sample Celltype Time Experiment
2-2 4481850214_B_Grn.idat    2-2       ML  At1          1
3-3 4481850214_C_Grn.idat    3-3       LP  At1          1
4-4 4481850214_D_Grn.idat    4-4    Basal  At1          1
6-7 4481850214_F_Grn.idat    6-7       ML  At2          1
7-8 4481850187_A_Grn.idat    7-8       LP  At2          1
8-9 4481850187_B_Grn.idat    8-9    Basal  At2          1
> experiment = as.character(data1$targets$Experiment)
> design = model.matrix(~0 + group + experiment)
> colnames(design) = gsub("group", "", colnames(design))
> design
   Basal LP ML experiment2
1      0  0  1           0
2      0  1  0           0
3      1  0  0           0
4      0  0  1           0
5      0  1  0           0
6      1  0  0           0
7      0  0  1           0
8      0  1  0           0
9      1  0  0           0
10     0  0  1           1
11     0  1  0           1
12     1  0  0           1
13     1  0  0           1
14     0  0  1           1
15     0  1  0           1
attr(,"assign")
[1] 1 1 1 2
attr(,"contrasts")
attr(,"contrasts")$group
[1] "contr.treatment"


                    attr(,"contrasts")$experiment
[1] "contr.treatment"


                    > contr.matrix = makeContrasts(
+         BasalvsLP = Basal-LP,
+         BasalvsML = Basal-ML,
+         LPvsML = LP-ML,
+         levels = colnames(design))
> contr.matrix
             Contrasts
Levels        BasalvsLP BasalvsML LPvsML
  Basal               1         1      0
  LP                 -1         0      1
  ML                  0        -1     -1
  experiment2         0         0      0
                


## 1. Creating gene set collection indexes

We next extract the mouse c2, c5 and KEGG gene signature collections from the **EGSEAdata** package and build indexes based on Entrez IDs that link between the genes in each signature and the rows of our expression matrix.



                    > library(EGSEA)
> library(EGSEAdata)
> gs.annots = buildIdx(entrezIDs=probe.annot[, 2],
+             species="mouse",
+             msigdb.gsets=c("c2", "c5"), go.part = TRUE)
[1] "Loading MSigDB Gene Sets ... "
[1] "Loaded gene sets for the collection c2 ..."
[1] "Indexed the collection c2 ..."
[1] "Created annotation for the collection c2 ..."
[1] "Loaded gene sets for the collection c5 ..."
[1] "Indexed the collection c5 ..."
[1] "Created annotation for the collection c5 ..."
MSigDB c5 gene set collection has been partitioned into
c5BP, c5CC, c5MF
[1] "Building KEGG pathways annotation object ... "
> names(gs.annots)
[1] "c2"   "c5BP" "c5CC" "c5MF" "kegg"
                


## 2. Configuring and 3. Testing with EGSEA

The same 11 base methods used previously in the RNA-seq analysis were selected for the ensemble testing of the microarray data using the function egsea.ma. Gene sets were again prioritised by their median rank across the 11 methods.



                    > baseMethods = egsea.base()[-2]
> baseMethods
 [1] "camera"     "safe"       "gage"       "padog"       "plage"       "zscore"
 [7] "gsva"       "ssgsea"     "globaltest" "ora"         "fry"
>
> gsam = egsea.ma(expr=expr, group=group,
+     probe.annot = probe.annot,
+     design = design,
+         contrasts=contr.matrix,
+         gs.annots=gs.annots,
+         baseGSEAs=baseMethods, sort.by="med.rank",
+         num.threads = 8, report = FALSE)
EGSEA analysis has started
##------ Tue Jun 20 14:27:32 2017 ------##
Log fold changes are estimated using limma package ...
limma DE analysis is carried out ...
Number of used cores has changed to 3
in order to avoid CPU overloading.
EGSEA is running on the provided data and c2 collection

EGSEA is running on the provided data and c5BP collection

EGSEA is running on the provided data and c5CC collection

EGSEA is running on the provided data and c5MF collection

EGSEA is running on the provided data and kegg collection

##------ Tue Jun 20 14:33:37 2017 ------##
EGSEA analysis took 365.359 seconds.
EGSEA analysis has completed
                


## 4. Reporting EGSEA results

An HTML report that includes each of the gene set level and summary level plots shown individually for the RNA-seq analysis was then created using the generateReport function. We complete our analysis by displaying the top ranked sets for the c2 collection from a comparative analysis across all contrasts.



                    > generateReport(gsam, number = 20, report.dir="./mam-ma-egsea-report")
EGSEA HTML report is being generated ...
> topSets(gsam, gs.label="c2", contrast = "comparison", names.only=TRUE, number=5)
 Sorted by med.rank
 [1] "LIM_MAMMARY_STEM_CELL_UP"
 [2] "LIM_MAMMARY_LUMINAL_MATURE_DN"
 [3] "LIM_MAMMARY_STEM_CELL_DN"
 [4] "CHARAFE_BREAST_CANCER_LUMINAL_VS_MESENCHYMAL_DN"
 [5] "LIU_PROSTATE_CANCER_DN"
                


The EGSEA report generated for this dataset is available online at http://bioinf.wehi.edu.au/EGSEA/mam-ma-egsea-report/index.html. Reanalysis of this data retrieves similar c2 gene sets to those identified by analysis of RNA-seq data. These included the LIM gene signatures (sets 1, 2 and 3) as well as those derived from populations with similar cellular origin (set 4).

## Discussion

In this workflow article, we have demonstrated how to use the **EGSEA** package to combine the results obtained from different gene signature databases across multiple GSE methods to find an ensemble solution. A key benefit of an EGSEA analysis is the detailed and comprehensive HTML report that can be shared with collaborators to help them interpret their data. This report includes tables prioritising gene signatures according to the user specified analysis options, and both gene set specific and summary graphics, each of which can be generated individually using specific R commands. The approach taken by EGSEA is facilitated by the diverse range of gene set testing algorithms and plotting capabilities available within Bioconductor. EGSEA has been tailored to suit a limma-based differential expression analysis which continues to be a very popular and flexible platform for transcriptomic data. Analysts who choose an individual GSE algorithm to prioritise their results rather than an ensemble solution can still benefit from EGSEA’s comprehensive reporting capability.

## Software availability

Code to perform this analysis can be found in the **EGSEA123** workflow package available from Bioconductor: https://www.bioconductor.org/help/workflows/EGSEA123.

Latest source code is available at: https://github.com/mritchie/EGSEA123.

Archived source code as at the time of publication is available at: https://doi.org/10.5281/zenodo.1043436 ^40^.

Software license: Artistic License 2.0.
