# Supplementary material for: Easy and efficient ensemble gene set testing with EGSEA
Source: F1000Res. 2017 Nov 14;6:2010. [Version 1] doi: 10.12688/f1000research.12544.1 (PMC5747338; doi:10.12688/f1000research.12544.1)
Supplement: Peer review history

# Peer review: Referee response for version 1

**DOI:** 10.5256/f1000research.13583.r27967

**Published:** 2017-12-20

**Authors:** Pekka Kohonen, Roland Grafström

**Affiliations:** 1 Institute of Environmental Medicine, Karolinska Institutet, Stockholm, Sweden; 2 IMM Institute of Environmental Medicine, Karolinska Institutet, Stockholm, Sweden

GSEA analysis methods do not all produce same results. Score-based gene set analysis methods like the Broad Institute GSEA tool are considered to perform better than normal Fisher’s exact test (overrepresentation analysis). But analysts often use methods they know to be less than ideal in order to reduce complexity and save time. So it is good to have a unified interface for GSEA analyses with R – it helps save programming time and reduces complexity. In addition EGSEA is a unique method that combines up to 12 gene set analysis methods into a single score. Independent test also corroborate that the tool using the 12 has more specificity and good sensitivity compared to using some of the tests alone.

 The EGSEA 1-2-3 workflow is easy to use and generate good-quality figures with the ggplot2 R package. So9me of the figures are novel compared to other packages e.g., scatter plots designed to compare different contrasts. It is also very useful that the tool can be applied to multiple contrasts at a time, although if there are too many contrasts then the number of plots becomes unwieldly (increases combinatorically).

 Some more technical comments:

 The results object is very complicated for retrieving individual method analysis results (although summaries are readily available). I quite like the "biobroom" Bioconductor package that does "tidy" data frames from limma results objects.

 All in all a very useful package both for automating the running of lots of methods at the same time and of course for the "ensemble" method.  It is recommended to be considered to be part of a standard bioinformatics workflow.

We have read this submission. We believe that we have an appropriate level of expertise to confirm that it is of an acceptable scientific standard.

# Peer review: Referee response for version 1

**DOI:** 10.5256/f1000research.13583.r27970

**Published:** 2017-12-13

**Authors:** Weijun Luo

**Affiliations:** 1 Department of Bioinformatics and Genomics, UNC Charlotte (University of North Carolina at Charlotte), Charlotte, NC, USA

EGSEA is a new gene set analysis tool that combines results from multiple individual tools in R as to yield better results. The authors have published EGSEA methodology previously. This paper focuses on the practical analysis workflow based on EGSEA with specific examples. As EGSEA is a compound and complicated analysis procedure, this work serves as a valuable guidance for the users to make full use of this tool. I’ve gone through the workflow line by line, it seems to work well. However, authors can improve their work by addressing the following issues.

  

1. There should be an R code script which includes all source code and concise comments like the one in company with the vignette in any Bioconductor package. It would be much easy for the users/reviewers to try the example code. It is not convenient to follow the code in this manuscript, the code need to be edit to remove the prompt symbols (> or +) at each line when copying/pasting.
2. It takes too long to run the egsea analysis example on modest machine. It is advisable to show a lesser example in the workflow with only one gene set collection like kegg and just a few base methods like:
  gsa = egsea(voom.results=v, contrasts=contr.matrix, 
           gs.annots=gs.annots$kegg, symbolsMap=symbolsMap,
           baseGSEAs=baseMethods[1:4], sort.by="med.rank",
           num.threads = 3, report = FALSE)
3. The rank of the gsa results shown following the t = topSets(..) line is confusing. The p.adj for the top 1 gene set is not the smallest, actually much bigger than top 2, 6 and 8. Presumably, the gene sets are ranked by med.rank instead of p.adj here. However, the opposite was described in the text above near the egsea.sort() line: “Although p.adj is the default option for sorting EGSEA results for convenience, ...”
4. In addition, there is big difference between the final rank and med.rank (e.g. 1 vs 36). This may suggest inconsistent results came from different base methods. This may also be due to the large number of gene sets being tested. Again, using a smaller gene set collection and a few base methods could make the ranking more consistent.
5. All visualization functions, i.e. plotHeatmap, plotPathway, plotGOGraph, plotMethods, plotSummary and plotBars share largely the same set of arguments, they can have a unified wrapper function like plot.gsa() with an extra argument type to specify the plot type.
6. Functions plotPathway, plotGOGraph are wrapper functions for those in the pathview and topGO package as the author noted in the text. It would be good to explicit show some message like “calling plotting function from pathview or topGO package etc”, just like the message when running egsea().
7. HTML report of the results is a very valuable feature for the users. However, the code can run a long time, it would be helpful to add some progress reminder message to generateReport() function like egsea(). BTW, the KEGG Pathway graphs are not shown properly in the report example at http://bioinf.wehi.edu.au/EGSEA/mam-rnaseq-egsea-report/index.html.

I have read this submission. I believe that I have an appropriate level of expertise to confirm that it is of an acceptable scientific standard, however I have significant reservations, as outlined above.

# Peer review: Referee response for version 1

**DOI:** 10.5256/f1000research.13583.r27974

**Published:** 2017-12-05

**Authors:** Jenny Drnevich

**Affiliations:** 1 Roy J. Carver Biotechnology Center, University of Illinois at Urbana–Champaign, Urbana, IL, USA

This F1000 software tool article describes the EGSEA package that incorporates many different gene set testing methods from various packages and also allows access to a wide array of gene sets from different databases through the accompanying EGSEAdata package. These packages will enable researchers to conveniently test many different methods and incorporate their results to get more robust biological insights ^1^, and this article gives a well-written walk-through of how to use the packages.

 The biggest limitation I see it that EGSEA is focused only on human and mouse data (and rat? The article does not list rat but the help page for buildIdx() lists rat as one of the species). I understand that many of the gene set collections like MSigDB and GeneSetDB are only available for human/mouse, but KEGG currently lists 429 Eukaryotic organisms ( http://www.genome.jp/kegg/catalog/org_list.html) and GO terms are readily available for 19 species using BioC's pre-built OrgDB packages and hundreds of other through AnnotationHub. It is unclear whether EGSEA functions buildCustomIdx and buildGMTIdx that were "written to allow users to run EGSEA on gene set collections that may have been curated within a lab or downloaded from public databases and allow use of gene identifiers other than Entrez IDs" can be used to run EGSEA on additional species. If so, this should be clearly stated in both the Abstract and in the body of the article, plus an example given on how to use buildCustomIdx for another species. If there is some reason that EGSEA cannot currently extend to other species, this should be acknowledged as a limitation and future versions should strive to allow this (although not required before approval). 

 Other issues to address before approval:

1. I am unable to create the html report on my Windows machine, getting the following error:
  Build GO DAG topology ..........
  There are no adj nodes for node: GO:0061857
  Error in switch(type, isa = 0, partof = 1, -1) : 
  EXPR must be a length 1 vector
  However, I reported this error to the support site ( https://support.bioconductor.org/p/103640/#103748)  and got a speedy reply from the author. It hopefully will be resolved soon, although there is a concern of why the error was not found on another Windows machine. 
2. I am concerned that as demonstrated in this paper, EGSEA seems to take the place of standard limma differential expression analysis, in that the model fitting takes place within the egsea() function. Certain gene set testing functions do need the individual expression values and not just the fitted values in an MArrayLM object but given the computational time (8 min as shown in the article code block and 19 min on my own computer) you should never run egsea() without first assessing the model fit on your own! Ideally the egsea function could be written to accept MArrayLM, or at least the article should clearly state that users should have first assessed the validity of the model fit through the usual workflow of Law *et al. *(2016) ^2^ prior to running EGSEA.
3. I also wonder why there are different interfaces for voom-based analysis and microarray data given that both use EList objects. I understand that the voom weights need to be used internally, but limma's lmFit function handles both without trouble, although it was originally coded for microarray data and the voom functionality came later. Even if there needs to be a separate function egsea.ma() for non-voom, non-count data, it should still accept an EList object so that the user does not have to pull out the expression data and the grouping info.
4. Back to the computational time required, there are several vague references to removing the roast method "to save time" and that the report generation "significantly" increases run time. it would be nice to have an example of the time required to run roast and the report generation for the computational architecture that created the article. 

I have read this submission. I believe that I have an appropriate level of expertise to confirm that it is of an acceptable scientific standard, however I have significant reservations, as outlined above.

## References

[1] AlhamdooshMNgMWilsonNSheridanJHuynhHWilsonMRitchieM: Combining multiple tools outperforms individual methods in gene set enrichment analyses. *Bioinformatics*.2016; 10.1093/bioinformatics/btw623 10.1093/bioinformatics/btw623 27694195PMC5408797

[2] LawCWAlhamdooshMSuSSmythGKRitchieME: RNA-seq analysis is easy as 1-2-3 with limma, Glimma and edgeR. *F1000Res*.2016;5: 10.12688/f1000research.9005.2 1408 10.12688/f1000research.9005.2 27441086PMC4937821

# Peer review: Referee response for version 1

**DOI:** 10.5256/f1000research.13583.r27972

**Published:** 2017-11-27

**Authors:** Robert Castelo

**Affiliations:** 1 Department of Experimental and Health Sciences, Universitat Pompeu Fabra, Barcelona, Spain

This article describes a gene set enrichment analysis (GSEA) workflow for the "Ensembl of GSEA" (EGSEA) R/Bioconductor software package.  EGSEA is an ensemble-like method recently published ^1^ by the authors of this workflow that allows the user to simultaneously apply different GSEA algorithms on a high-throughput molecular profiling data set, by combining p-values associated with each algorithm using classical meta-analysis approaches such as the Fisher's method.

 Because the statistical methodology is already described in detail in the corresponding publication, the present software tool article focuses on showing a step-by-step workflow with EGSEA. However, the vignette of the software package already provides a very detailed description about how to use EGSEA through its 39 pages. Therefore, it would be useful for the interested reader to find upfront when he/she should be consulting the vignette and when he/she should be consulting this workflow. Besides this introductory aspects, the following issues should be addressed before approval:

  

1. The code given in the article breaks, at least in my computer, more concretely, at this line:
  gsa = egsea(voom.results=v, contrasts=contr.matrix,
              gs.annots=gs.annots, symbolsMap=symbolsMap,
              baseGSEAs=baseMethods, sort.by="med.rank",
              num.threads = 8, report = FALSE)
  EGSEA analysis has started
  ##------ Mon Nov 27 12:37:42 2017 ------##
  Log fold changes are estimated using limma package ...
  limma DE analysis is carried out ...
  Number of used cores has changed to 4
  in order to avoid CPU overloading.
  EGSEA is running on the provided data and c2 collection
  .......camera*....safe*...gage*.padog*....gsva*..fry*...plage*...globaltest*...zscore*...ora*...ssgsea*
    Error in temp.results[[baseGSEA]][[i]][names(gs.annot@idx), ] :
    incorrect number of dimensions
  while running it with the latest release version 1.6.0. This is strange since the package builds and runs the vignette without problems. So, this might be related to the different sample data sets. A possible hint may come from the fact that the 'buildIdx()' call is not returning the expected class of object, according to the workflow:
  class(gs.annots$s2)
  ## [1] "NULL"
  summary(gs.annots$s2)
  ## Length  Class   Mode 
  ##      0   NULL   NULL 
2. The workflow contains a rather high amount of code, often with a non-trivial use of externally instantiated objects and nested calls to functions. It would be helpful for the interested reader to be able to easily copy and paste the instructions, but the fact that R commands are given with the R shell '>' and '+' symbols makes it less easy. A non-expert user may even copy those characters and get an error. I would recommend removing those characters from the illustrated code, just as it happens with the vignette.
3. The workflow assumes that the user has a 'DGEList' object with gene metadata including the mapping between Entrez identifiers' and HGNC symbols. This is a rather unrealistic assumption and I would recommend that the workflow starts building that object from scratch and showing how to build that table of gene metadata.

Below I also describe other issues that I would recommend to be considered in future versions of the software but which I do not consider them to be required for approval of this article:

1. The so-called "summary plot" shows the -log10 p-value on the x-axis and average absolute log fold-change of the set genes on the y-axis. Because this is in a way analogous to a rotated volcano plot, I would suggest to use the same arrangement of axes as in the volcano plot, which is a rather standardized display of significance and magnitude of the effects of interest.
2. One of the key features of the Bioconductor project, to which the EGSEA package is contributing to, is enabling software interoperability through sharing the use of common data structures across different software packages.  Using specialized data structures, where analogous ones have been already designed by the Bioconductor core team or by a wider community of developers, locks the user into that package and limits the possibilities of using it as a building block in other more complex workflows. I'm making this comment because I have the impression that the EGSEA package would benefit of using the infrastructure provided by the Bioconductor GSEABase package, in which data structures are defined to store and access gene sets and collections of gene sets of different kinds. A salient feature of that infrastructure is the possibility to seamlessly map gene identifiers of different kinds. This would simplify and improve the user experience of EGSEA since mapping between genes coded with a particular kind of identifier, and gene sets defined with another kind, is one of the most common tasks in a GSEA-like analysis.

I have read this submission. I believe that I have an appropriate level of expertise to confirm that it is of an acceptable scientific standard, however I have significant reservations, as outlined above.

## References

[1] AlhamdooshMNgMWilsonNSheridanJHuynhHWilsonMRitchieM: Combining multiple tools outperforms individual methods in gene set enrichment analyses. *Bioinformatics*.2016; 10.1093/bioinformatics/btw623 10.1093/bioinformatics/btw623 27694195PMC5408797
